# Analysis of effector/memory regulatory T cells from arrhythmogenic cardiomyopathy patients identified IL-32 as a novel player in ACM pathogenesis

**DOI:** 10.1038/s41419-025-07364-y

**Published:** 2025-02-11

**Authors:** Salwa Soussi, Angela Serena Maione, Lise Lefèvre, Nathalie Pizzinat, Jason Iacovoni, Ignacio Gonzalez-Fuentes, Daniel Cussac, Lara Iengo, Yohan Santin, Fabrizio Tundo, Claudio Tondo, Giulio Pompilio, Angelo Parini, Victorine Douin-Echinard, Elena Sommariva

**Affiliations:** 1 I2MC, INSERM, UMR-1297, Toulouse, France; 2 Unit of Inherited Cardiomyopathies, Centro Cardiologico Monzino IRCCS, 20138 Milan, Italy; 3 RESTORE Research Center, UMR-1301, Paul Sabatier University, Toulouse, France; 4 Department of Biomedical Sciences for Health, Università degli Studi di Milano, Milan, Italy; 5 Department of Clinical Electrophysiology and Cardiac Pacing, Centro Cardiologico Monzino IRCCS, 20138 Milan, Italy; 6 Department of Biomedical, Surgical and Dental Sciences, Università degli Studi di Milano, Milan, Italy

**Keywords:** Cardiomyopathies, Cell biology

## Abstract

Arrhythmogenic cardiomyopathy (ACM) is an inherited cardiac disorder that causes sudden cardiac death and progressive heart failure. Besides fibro-fatty replacement and myocyte degenerative changes, inflammatory patchy infiltrates are found in myocardial histological analysis of ACM patients. Inflammatory cells could actively participate in ACM pathogenesis, contributing to the alteration of cardiac microenvironment homeostasis, thus triggering disease evolution. In order to characterize the immune-derived mediators involved in ACM pathogenesis, peripheral blood mononuclear cells from ACM patients were characterized and compared to healthy controls’ ones. Flow cytometry analysis revealed a lower frequency of CD4^+^ T helper type 1 cells, NK cells, and terminally differentiated CD8^+^ EMRA^+^ T cells in ACM patients compared to age-matched controls. In contrast, a higher proportion of effector/memory FOXP3^+^ CCR4^+^ CD45RO^+^ regulatory CD4^+^ T cells (Treg) were found in ACM patients. Single-cell RNA-seq performed on isolated memory Treg cells (mTreg) from ACM patients and healthy controls identified 6 clusters characterized by specific gene signatures related to tissue repair and immunosuppressive pathways. Notably, interleukin 32 (IL-32) was the most differentially expressed gene in ACM patients mTreg with respect to healthy controls. Treatment of human cardiac mesenchymal stromal cells with recombinant IL-32 in vitro promoted lipid droplet accumulation and collagen deposition, thus identifying IL-32 as a new potential player in the immune-mediated trigger of cardiac fibro-fatty replacement in ACM. Overall, we here provide the first complete characterization of circulating ACM immune cells, revealing an abundance of Treg. The high expression of IL-32 in ACM Treg may contribute to accelerated cardiac remodeling in ACM patients’ hearts.

## Introduction

Arrhythmogenic Cardiomyopathy (ACM) is an inherited heart disease that causes sudden cardiac death and progressive heart failure (HF) in young people, particularly those who are athletes [1]. In most cases, it is caused by autosomal dominant mutations in genes encoding desmosomal proteins, such as plakophilin-2 (*PKP2*), desmoplakin (*DSP*), plakoglobin (*JUP*), desmocollin-2 (*DSC2*) or desmoglein-2 (*DSG2*) [2]. These proteins are part of the intercalated disc complex which interconnects cardiomyocytes (CM) and are essential for intercellular electromechanical coupling and intracellular signaling pathways [3]. Hearts of ACM patients exhibit extensive CM death and fibrofatty tissue replacement mainly in the right ventricle (RV), primarily imputable to cardiac stromal cell differentiation (C-MSC) [4, 5]. This creates electrically heterogeneous tissue that contributes to arrhythmogenesis and progressive contractile impairment, mainly in the right ventricle [6].

Inflammation has long been recognized as a characteristic of ACM, and it is frequently observed in differential diagnoses with myocarditis [7, 8]. Patchy inflammatory infiltrates are consistently reported in ACM heart biopsies, and autoptic cases [9–12]. They are more frequent in patients with advanced phases of the disease, involving the left ventricle [13]. Since the presence of inflammatory infiltrates is evident in areas with severe structural heart remodeling, it has been proposed that it can modulate ACM severity [14]. Neutrophils, T lymphocytes, and macrophages are the most common inflammatory cells present in the cardiac blood vessels or around them [14] hinting at recruitment to the heart and activation from the circulatory system.

Cardiomyocyte death in the context of desmosomal dysfunctions was believed to be the initial event of ACM, which is fueled by an inflammatory response of variable magnitude. However, recent reports highlighted the activation of the innate immune response in the heart as a primary driver mechanism of ACM, as ACM cardiac myocytes express large amounts of inflammatory cytokines and chemotactic molecules [15], recruiting immune cells to the heart [16]. Accordingly, plasma levels of proinflammatory cytokines (IL1-β, IL-6, and TNFα) and chemokines (CCL-2, CCL-3) [17, 18], as well as complement factors [19] were reported higher in ACM patients than in controls, suggesting a systemic para-inflammation [20]. Interestingly, autoantibodies are detected in ACM patients’ serum and autoimmunity is thought to play a role in ACM pathogenesis [21–23].

To date, the evidence of the role of innate and adaptive immune cells in regulating diverse functions to maintain cardiac homeostasis, both in physiological conditions and after pathological stressors, is growing [24]. In particular, the heart may benefit or be harmed by the enrichment of immune-cell subsets with different activation states. In response to cardiac injury, monocyte-derived CCR2^+^ macrophages have been shown to play a deleterious role in contributing to pathological cardiac remodeling in both mice and humans [25, 26]. Several studies have highlighted the role of cardiac macrophages and T lymphocytes in the onset and progression of cardiac dysfunction in both murine models and patients with HF [27, 28]. According to their effector functions, lymphocyte subsets impact the nature of cardiac remodeling Fig. 3.

Notably, the expansion of specific CD4^+^ T cell clonotypes in cardiac tissue during HF strongly supports the implication of an antigen-specific immune response [29, 30]. In inflammatory cardiomyopathies, circulating c-MET^+^ memory T cells have been described to home to the heart and participate in cardiac dysfunction [31]. Conversely, regulatory T cells (Treg) expressing CCR4 have been shown to exert a protective effect in response to barometric stress in mice [32, 33] and to have pro-resolving functions in tissues [34]. Patients with dilated cardiomyopathy have a reduced frequency of circulating CD4^+^ LAP^+^ Treg, possibly leading to chronic immune activation, and disease progression [35]. In the first steps of cardiac remodeling, cardiac Treg inhibit macrophage-dependent production of proinflammatory cytokines, leading to decreased fibrosis and cardiac hypertrophy. Conversely, at later stages of post-myocardial injury, Treg expressing TNFR1, secreting IFN-γ and TNF-α harboring anti-angiogenic functions and loss of repressive activity have been described [36].

To date, an extensive characterization of the circulating immune T cell subsets has never been performed in ACM patients. CD4^+^ T cells are potential sources of cytokines regulating fibrosis and adipogenesis (IL-4, TGFβ, IL-17, IL-6), however, modulation of C-MSC properties by immune-cell-derived cytokines has yet to be investigated.

The present work sheds light on the inflammatory status of ACM patients and identifies cellular and soluble mediators involved in the ACM-specific inflammatory cascade, possibly leading to cardiac fibro-adipogenesis.

## Results

### Profiling of immune-cell populations in the blood of ACM patients

**Fig. 1:**
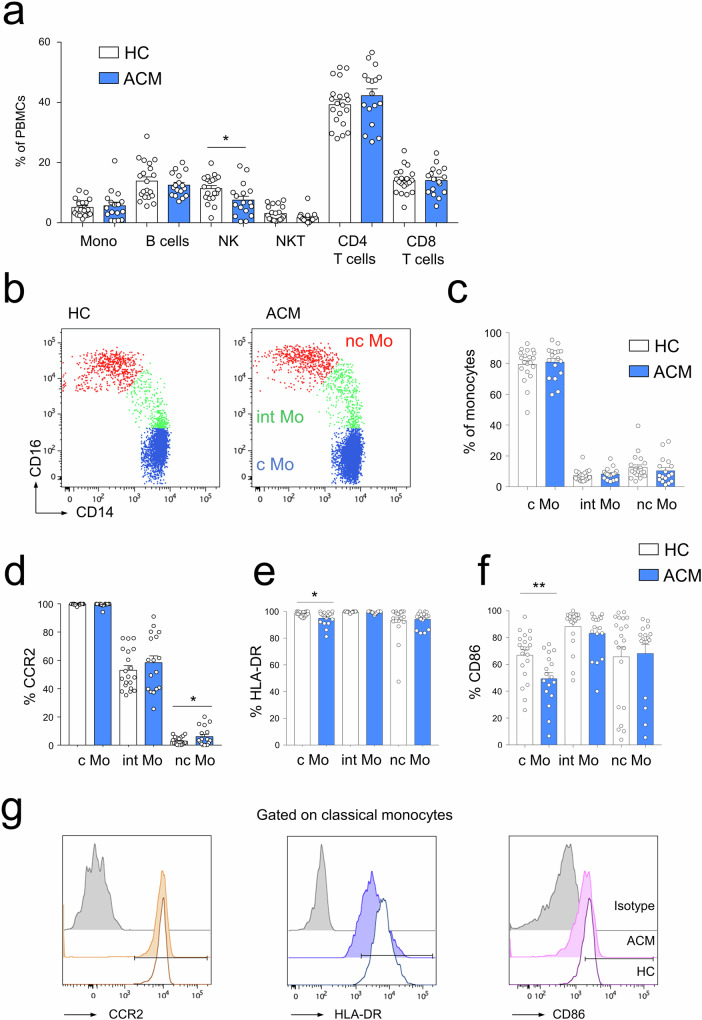
Analysis of immune cell populations from PBMC of ACM patients revealed decreased NK cell frequency and phenotypic changes of the classical monocyte subset. **a** Frequencies of major blood leukocyte subsets of HC (*n* = 20) and ACM patients (*n* = 17) analyzed by flow cytometry. *p < 0.05, **p < 0.01, and ns not significant using Unpaired *t*-test or Mann–Whitney *t*-test. **b** Blood monocyte subsets of controls and ACM patients were characterized by flow cytometry using CD14 and CD16 staining. **c** Histograms showing monocyte subset frequencies percentages of monocytes positive for **d** CCR2, **e** HLA-DR, and **f** CD86 for the monocyte subsets of healthy controls (HC; *n* = 24) and ACM patients (ACM; *n* = 20). **g** Representative fluorescence overlay histograms show CCR2, HLA-DR, and CD86 expression by classical monocytes of HC and ACM patients compared to isotype mAb staining (gray). *p < 0.05, **p < 0.01, and ns not significant using Unpaired *t*-test or Mann–Whitney *t*-test.

We first analyzed the frequencies of immune-cell lineages from the peripheral blood of ACM patients and age-matched healthy controls by flow cytometry (gating strategy, Fig. S1a). Monocytes, NKT cells, CD4 or CD8 T lymphocytes, and B cells had comparable frequencies except for natural killer (NK) cells, which were less abundant in ACM patients compared to controls (Fig. 1a).

Since changes in human blood monocyte subsets were reported in the context of various cardiovascular diseases and are well-known players of cardiac remodeling [37, 38], we monitored the frequencies of the three monocyte subsets based on CD14 and CD16 expression, defining classical monocytes (CD14^+^, CD16^–^ cMo), intermediate monocytes (CD14^+^, CD16^int^ intMo) and nonclassical monocytes (CD14^low^, CD16^high^ ncMo) (Fig. 1b). ACM patients showed no difference of monocyte subset frequencies compared to age-matched healthy controls (HC) (Fig. 1c). However, analysis of surface markers revealed that ncMo had higher expression of CCR2 (Fig. 1d, g) and the cMo subset lower HLA-DR (Fig. 1e, g) and CD86 (Fig. 1f, g) in ACM patients compared to HC. This lower expression of HLA-DR and costimulatory molecules by the cMo subset of ACM patients is evocative of impaired antigen presentation potency and monocyte anergy [39], usually triggered in response to acute or chronic inflammation [39, 40].

**Fig. 2:**
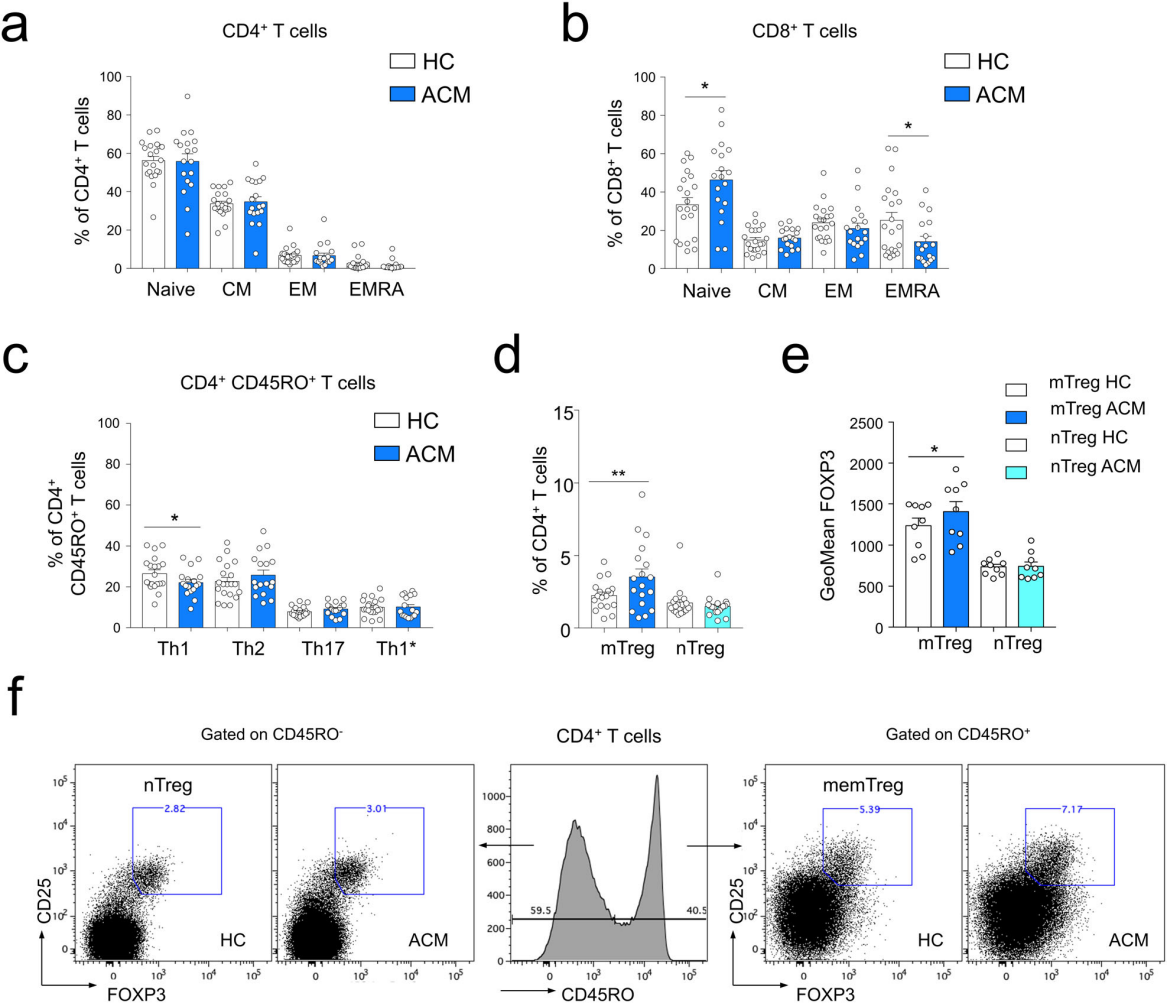
The analysis of CD4^+^ T cell subsets of ACM patients revealed blood effector/memory CD4^+^ T regulatory cell increase and effector-memory CD8^+^ cell decrease. **a**, **b** Percentages of naive, CM, EM, and EMRA subsets in CD4+ T cells (**a**) or in CD8+ T cells (**b**) from controls (*n* = 21) and ACM patients (*n* = 18) as histograms. **c** Percentages of memory CD45RO^+^ CD4^+^ T helper subsets Th1-like, Th2-like, Th17-like and Th1*-like from ACM patients and age-matched controls (HC; *n* = 17 pairs). **d**, **e** Percentages (*n* = 18 per group) of mTreg and naive (nTreg) in CD4^+^ T cells (**d**) and geometric mean fluorescence intensity (*n* = 9 per group) of FOXP3 (**e**). **f** Analysis of mTreg and nTreg CD4^+^ T regulatory cell subsets of HC and ACM patients by flow cytometry based on CD45RO expression. Data are shown as mean ± SEM. **p < 0.01, *p < 0.05 and ns not significant using unpaired *T*-test (**a**, **b**) or Wilcoxon matched-pairs signed rank test (**c**–**e**).

We hypothesized that mobilization of T cell responses during ACM pathogenesis could impact the relative distribution of T lymphocytes in the naive versus memory pool compared to age-matched healthy controls. Based on CCR7 and CD45RO differential expression, we analyzed the frequency of naive (CCR7^+^ CD45RO^−^), central memory (CM, CCR7^+^ CD45RO^+^), effector memory (EM, CCR7^−^ CD45RO^+^) and terminally differentiated memory (EMRA, CCR7^−^ CD45RO^−^) T cell subsets within the CD4^+^ and CD8^+^ T cell populations (Fig. S1b). The relative distribution of CD4^+^ T cells in the naive and memory pool was not significantly different in ACM patients compared to HC (Fig. 2a, Fig. S1b). Conversely, the naive CD8^+^ T cell pool was present at a higher percentage in ACM patients whereas the terminally differentiated memory EMRA CD8^+^ T cell subset had lower frequencies compared to HC (Fig. 2b, Fig. S1b), regardless of age (Fig. S1c, d) suggesting that conversion of naive CD8^+^ T cells toward the memory phenotype is impeded in ACM. We then asked if the modification of the memory CD8^+^ T cell pool in ACM patients was associated with specific changes in the memory helper CD4^+^ T cell subsets. Using the expression profile of the chemokine receptors CXCR3, CCR4 and CCR6, we assessed the relative frequency of Th1-like CD4^+^ T cells (CCR6^−^ CCR4^−^ CXCR3^+^), Th2-like CD4^+^ T cells (CCR6^−^ CCR4^+^ CXCR3^−^), Th17-like (CCR6^+^ CCR4^+^ CXCR3^−^) and alternative Th1*-like CD4^+^ T cells (CCR6^+^ CCR4^−^ CXCR3^+^) among memory CD4^+^ T cells (Fig. S2a). ACM patients showed lower frequency of the Th1-like subset compared to age-matched controls whereas no significant difference was observed for the other effector-memory CD4^+^ T cell subsets (Fig. 2c). Gene expression of master transcription factors associated with the effector functions of Type 1 (*TBX21*) or Type 2 (*GATA3*) helper memory CD4^+^ T cells showed lower expression of *TBX21* but not *GATA3* in ACM patients compared to HC (Fig. S2b). In addition, Fig. S2c–f confirmed that Th2 CD4^+^ T cells were not higher in ACM patients, regardless of age.

In ACM patients, the lower abundance of Th1 cells, NK cells, and EMRA CD8^+^ T cells, suggested that a compensatory immunosuppressive tone was orchestrated, possibly by Treg. To test this hypothesis, we first evaluated the percentage of CD4^+^ T regulatory cells (Treg, FOXP3^+^ CD25^+^) with a naive (CD45RO^−^ nTreg) or activated/memory (CD45RO^+^ mTreg) phenotype in ACM patients by flow cytometry (Fig. 2d–f). Naive Treg showed comparable percentages between ACM and HC with a similar expression of FOXP3 (Fig. 2d–f). Conversely, in ACM patients, the percentage of mTreg were significantly increased expressing higher FOXP3 compared to controls (Fig. 2d–f), with the same expression of CCR4^high^ and ICOS^high^ (Fig. S3a–c), two classical mTreg markers [41–43].

### Single-cell RNA-seq of memory Treg of ACM patients and HC

In order to explore the transcriptional profile of circulating activated/effector-memory Treg of ACM patients, we cell-sorted CCR4^+^ CD25^+^ CD45RO^+^ CD4^+^ T cells from PBMC of five *PKP2* mutated patients (PKP2*) and of five age- and sex-paired controls, and quantified gene expression using droplet-based scRNA-seq. This strategy enables the enrichment of different blood CCR4^+^ activated/memory Treg subtypes such as Th17-like (CCR4^+^CCR6^+^CXCR3^−^), Th1-like (CCR4^+^CCR6^−^CXCR3^+^) and Th2-like (CCR4^+^CCR6^−^CXCR3^−^) mTreg [44]. In total, we profiled 7857 cells, 4061 cells from controls, and 3796 cells from ACM patients, multiplexing individual samples in the same scRNA-seq libraries using DNA code tags [45]. Accordingly, using the Database of Immune Cell Expression (DICE) [46], we identified most of the analyzed cells as mTreg in both HC (2781) and ACM (2752), while few naive Treg and effector-memory CD4^+^T cells, mainly Th2 and Th17 cells, could be identified (Fig. S4a–c).

**Fig. 3:**
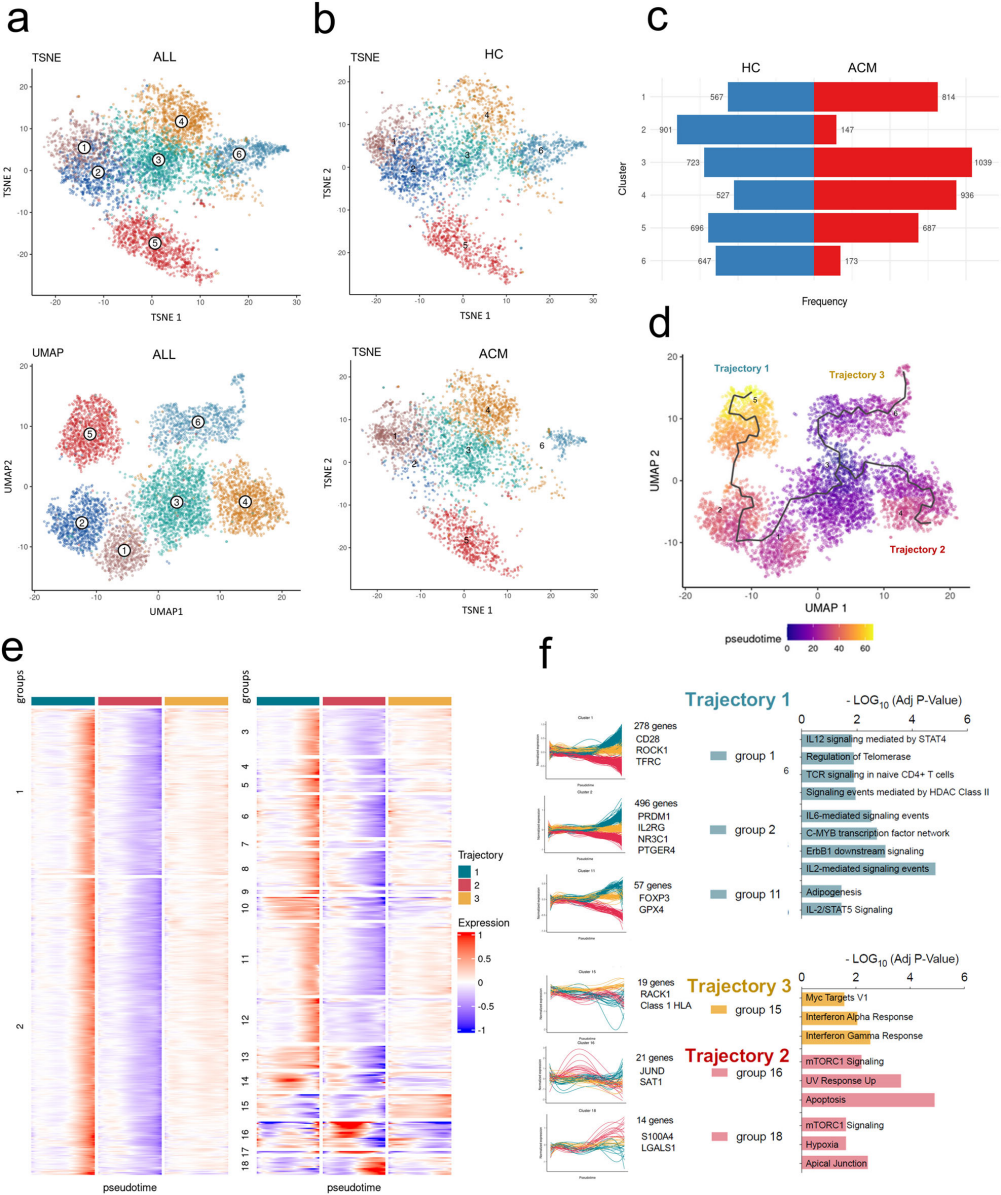
Single cell RNA-seq analysis of effector/memory CD4^+^ T cell reveal different subsets enrichment in ACM patients vs. HC. **a***t*-SNE and UMAP projections of single-cell transcriptomes of cell-sorted CD45RO^+^ CD25^+^ CCR4^+^ CD4^+^ mTreg from PBMC of ACM patients with PKP2* mutation and controls (*n* = 5 per group) show the presence of 6 clusters **b***t*-SNE projections of HC or ACM cells (separately) and **c** their respective contribution (frequency and cell numbers) to the 6 clusters. **d** Pseudotemporal gene-expression profiles of DICE selected mTreg defined three trajectories on a UMAP plot with cells colored according to its pseudotime. **e** Clustering of significant genes based on their expression pattern over pseudotime trajectories (|area between the curves|>0.5 with *p-*value < 0.01 in at least a pairwise comparison), defined 18 groups of genes. **f** Profiles of marker genes differentially expressed across the trajectories and pathway analysis related to groups of genes with similar expression patterns.

**Fig. 4:**
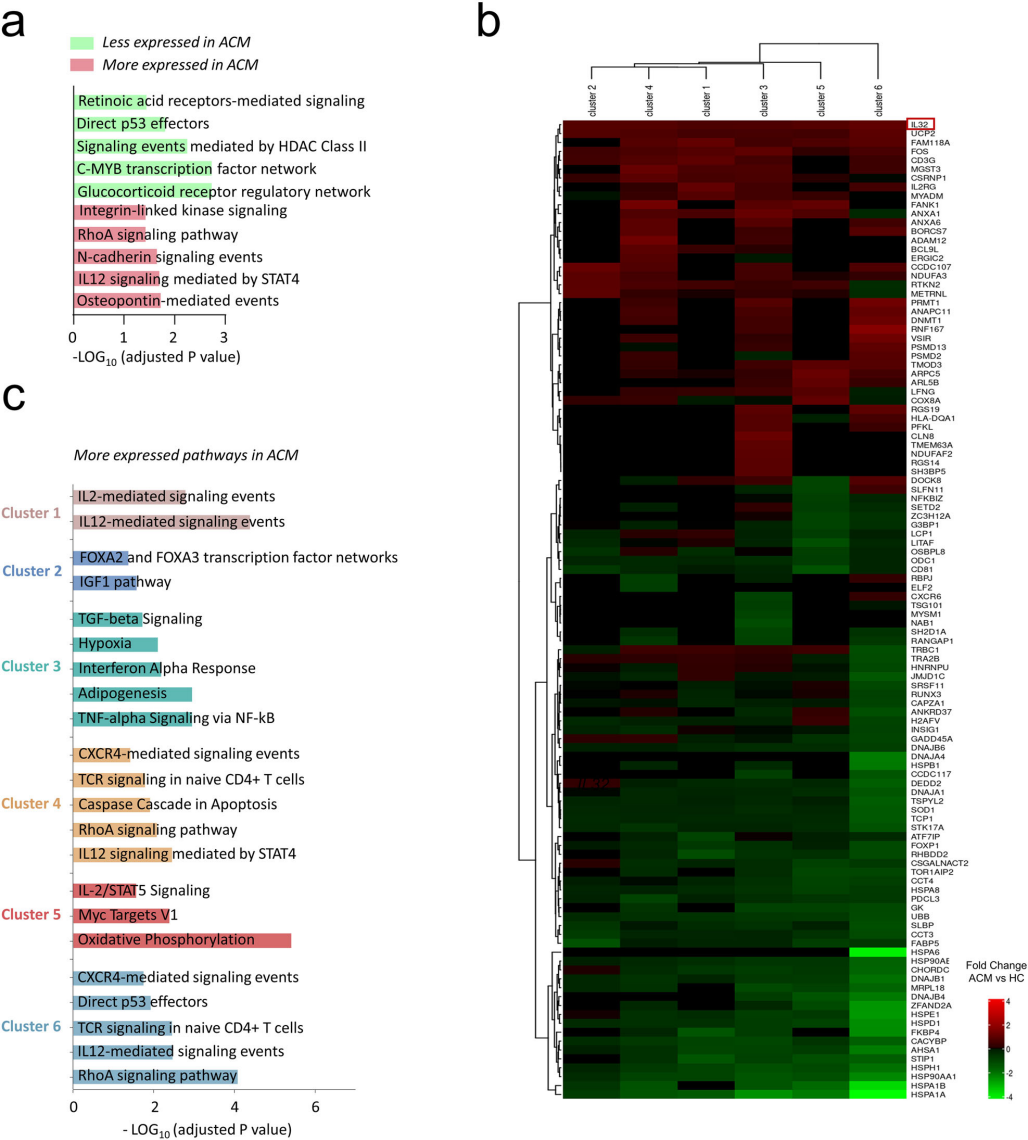
Differential expression analysis of Treg of ACM patients vs. HC revealed dysregulation of specific pathways and higher expression of *IL-32.* **a**, **c** Pathway analysis of differentially expressed genes between HC and ACM mTreg (**a**) and for each cluster (**c**), according to NCI-Nature Pathway Database. **b** Heatmap showing log-fold change expression of genes in each of the 6 clusters with significant ANOVA test (*p* > 0.01) between ACM and control identifying *IL-32*.

Seurat-based clustering method highlighted that memory CD4^+^ T cells from ACM patients and HC partitioned into 6 different clusters on the t-SNE and UMAP plots (Fig. 3a) with relative abundance depending on donor (ACM vs. HC) origin (Fig. 3b). While ACM and HC cells were equally represented in cluster 5, HC cells predominated in clusters 2 and 6, whereas ACM cells were more prevalent in clusters 1, 3, and 4. (Fig. 3b, c). Of note, expression of core Treg signature genes (*FOXP3*, *IL-2RA*, *IKZF2, ICOS,* and *CCR4*) overlapped the six clusters in HC and ACM cells, and reached the highest levels for cluster 5 (Fig. S4b), as memory CD4^+^ Treg cell subsets constitute a gradual population with different and overlapping cell states [47]. As detailed in the Supplementary Text and Fig. S5, using published Treg gene signatures [43, 48–52], we identified gene modules in the different clusters, associated with tissue residency (mostly in cluster 5, and very low in cluster 4), tissue repair (mostly in cluster 1), cell activation (mostly in cluster 6), effector functions and central memory (mostly in cluster 4). To explore the hierarchy relationship between clusters, pseudotime analysis was performed. The analysis identified three differentiation pathways emerging from cluster 3, with gene expression patterns over pseudotime displaying distinct profiles depending on the trajectory (Fig. 3d–f). Trajectory 1 progressed from cluster 1 to cluster 2, ultimately reaching cluster 5, following a differentiation path of effector/memory Treg characterized by tissue residency markers, such as the upregulation of *PRDM1, NR3C1, GPX4*, and *TFRC* (Fig. 3d–f). This trajectory was also linked to pathways suggesting TCR-dependent activation of resting memory Treg and FOXP3 upregulation, including “TCR-signaling in naive CD4^+^ T cells,” “IL-2/STAT5 signaling,” and “Signaling events mediated by HDAC Class II” [53] (Fig. 3d–f). Additionally, genes related to “IL-6 mediated signaling events,” important for muscle Treg maturation and tissue repair, were upregulated along this trajectory [54] (Fig. 3d–f). Trajectories 2 and 3 consisted of short paths from cluster 3 to cluster 4 or cluster 6 respectively (Fig. 3d). Path to cluster 4 (enriched in ACM cells) showed transit upregulation of early activation genes (*JUND, SAT1*) [55], escape from activation-induced cell death (“apoptosis”), and progressive expression of effector molecules (*S100A4, LGALS1*), with signaling pathways reinforcing their immunosuppressive functions (“mTORC1”, “Hypoxia”), likely following effector-memory differentiation pathway and adaptation to tissue hypoxia [56] (Fig. 3d–f). Path to cluster 6, enriched in HC cells, showed upregulated genes (class I HLA molecules) in “response to interferons” (Fig. 4b, c) highlighting a specialized interferon-dependent Treg activation pathway known as IFN^+^ Treg [55] (Fig. 3d–f).

The differential analysis of all mTreg between ACM and HC mTreg showed increased gene expression in ACM related to response to the proinflammatory cytokine “IL-12 signaling mediated by STAT4” and Th1 suppression (*IL-12RB1*), and to pro-fibrotic myocardial factors “Osteopontin-mediated events” described in ACM pathogenesis [57] (Fig. 4a). Gene upregulation in ACM mTreg also identified pathways governing cell motility and cell adhesion like “N-cadherin signaling events”, “Integrin-linked kinase signaling” and “RhoA signaling” described for tissue-Treg migration and mechanosensing (*TRPV2*) [56, 58] (Fig. 4a). Conversely, decreased gene expression by ACM mTreg identified alteration of key pathways regulating CCR8^+^ Treg infiltration and retention (“Glucocorticoid receptor regulatory network”) [59], Th17 suppression (“Retinoic acid receptors-mediated signaling”) and tissue specialization for Treg (“C-MYB transcription factor network”) [60], whereas decreased transcripts of the “p53-signaling pathway” could promote survival of activated mTreg in ACM patients (Fig. 4a). Differential gene expression analysis in each cluster (Fig. 4b and S6), and pathway analysis (Fig. 4c) showed that ACM was associated with mTreg activation in response to cytokines (TGFß, TNF-α IL-2, IL-12, IFN-γ) for clusters 1, 3 and 5 or to TCR engagement for cluster 4 and 6.

### IL-32 expression in PBMC, plasma, and right ventricle of ACM patients and HC

Next, we sought to identify specific transcripts for secreted factors in mTreg ACM relative to HC that might influence tissue repair and cardiac ACM remodeling. *IL-32* emerged as the most upregulated secreted factor in mem Treg ACM, consistently exhibiting higher levels across all clusters when compared to HC (Fig. 4b). To assess the expression levels and distribution of IL-32, we evaluated its expression in PBMC, plasma, and heart tissue from ACM patients and HC subjects.

**Fig. 5:**
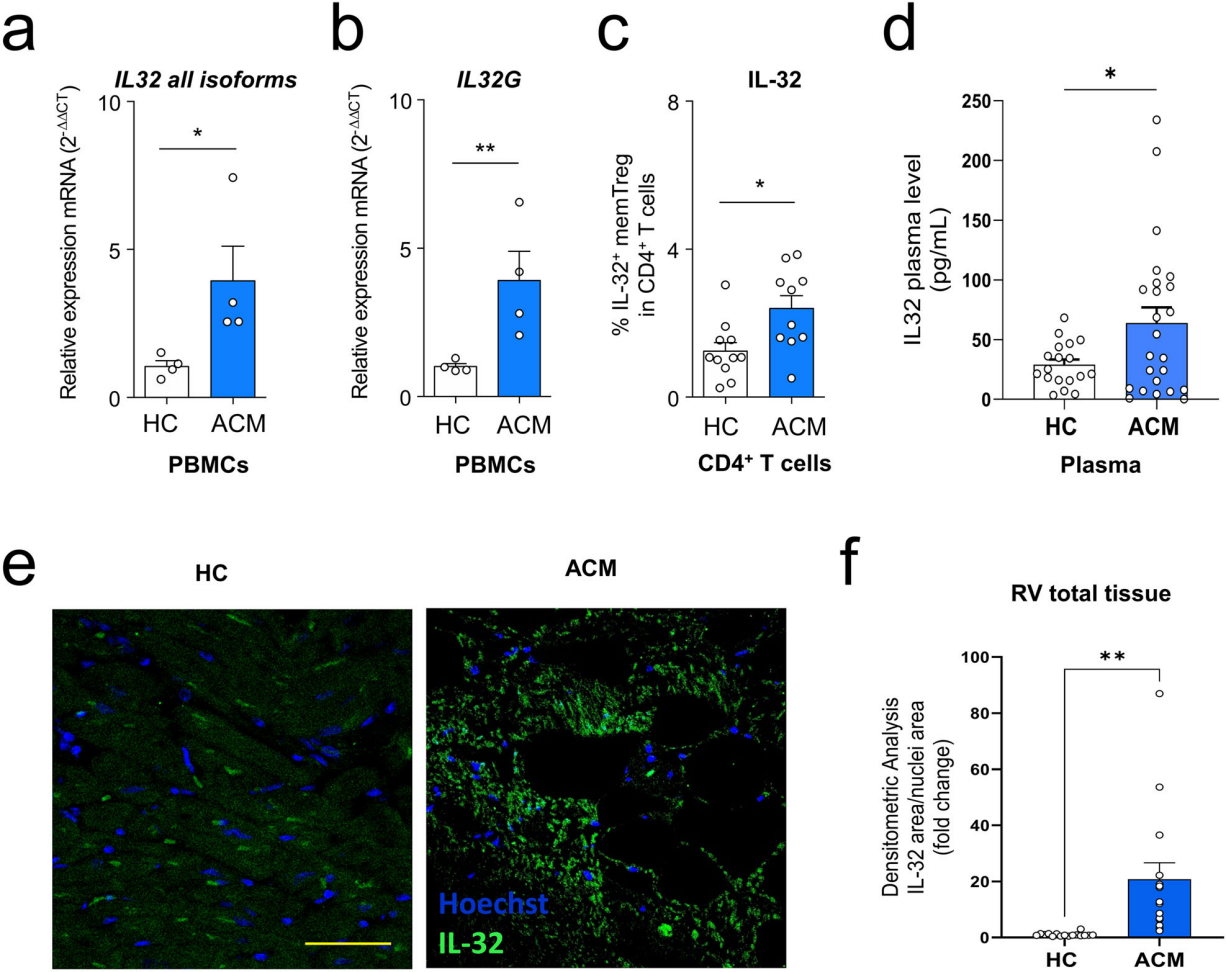
A higher expression of IL-32 characterizes plasma and right ventricle of ACM patients compared to HC. **a**, **b** Relative mRNA expression of total (**a**) and γ isoform (**b**) of the *IL-32* gene in PBMC from ACM patients or healthy donors (HC). Data were expressed using the 2^−^^ΔΔCt^ method over the housekeeping gene GAPDH. **c** Percentages of IL-32^+^ mTreg in CD4^+^ T cells from PBMC of ACM patients or healthy donors determined by flow cytometry after intracellular staining. **d** IL-32 concentration (pg/ml) in the plasma of ACM or HC (*n* = 24 each). **e**, **f** Representative images of right ventricular tissue sections from HC and ACM showing IL-32 staining by immunofluorescence (**e**) and histogram quantification (**f**), data are expressed as the total area of IL-32 positivity on nuclei area. Data are shown as mean +/− SEM. **p < 0.01, *p < 0.05 using *T*-test. Scale bar: 50 µm.

Levels of mRNA corresponding to all IL-32 variants or to the secreted isoform (IL-32 γ) were higher in ACM PBMC compared with HC ones (Fig. 5a, b), confirming the pattern observed in mTreg. Moreover, the frequency of effector Treg positive for IL-32 in the CD4^+^ T cell population was higher in ACM patients vs HC (Fig. 5c).

To determine if IL-32 was secreted from blood cells into the plasma, we performed an ELISA assay. Indeed, IL-32 was detected in all plasma samples analyzed, with significantly higher levels in ACM patients compared to HC (Fig. 5d).

We then investigated if we could detect IL-32 in the cardiac tissue and if there was a differential expression pattern between HC and ACM patients. To this aim, cardiac tissue biopsies were collected from ACM patients along with corresponding samples from control hearts, followed by immunofluorescence experiments. IL-32 resulted present at higher levels in the right ventricle tissue biopsies of ACM patients than in that of HC (Fig. 5e, f). In particular, IL-32 intense signal corresponded to CD45^+^ cells (Fig. S7a). Interestingly, reanalysis of the scRNA-seq dataset of cardiac cells from ACM PKP2* patients [61] revealed that the Tissue-Treg gene signature was upregulated in cardiac lymphocytes of ACM patients versus HC (Fig. S7b), with *IL-32* primarily expressed in leukocytes and lymphocytes of PKP2*-mutated hearts (Fig. S7c).

### Effect of IL-32 on C-MSC adipogenesis and fibrosis

**Fig. 6:**
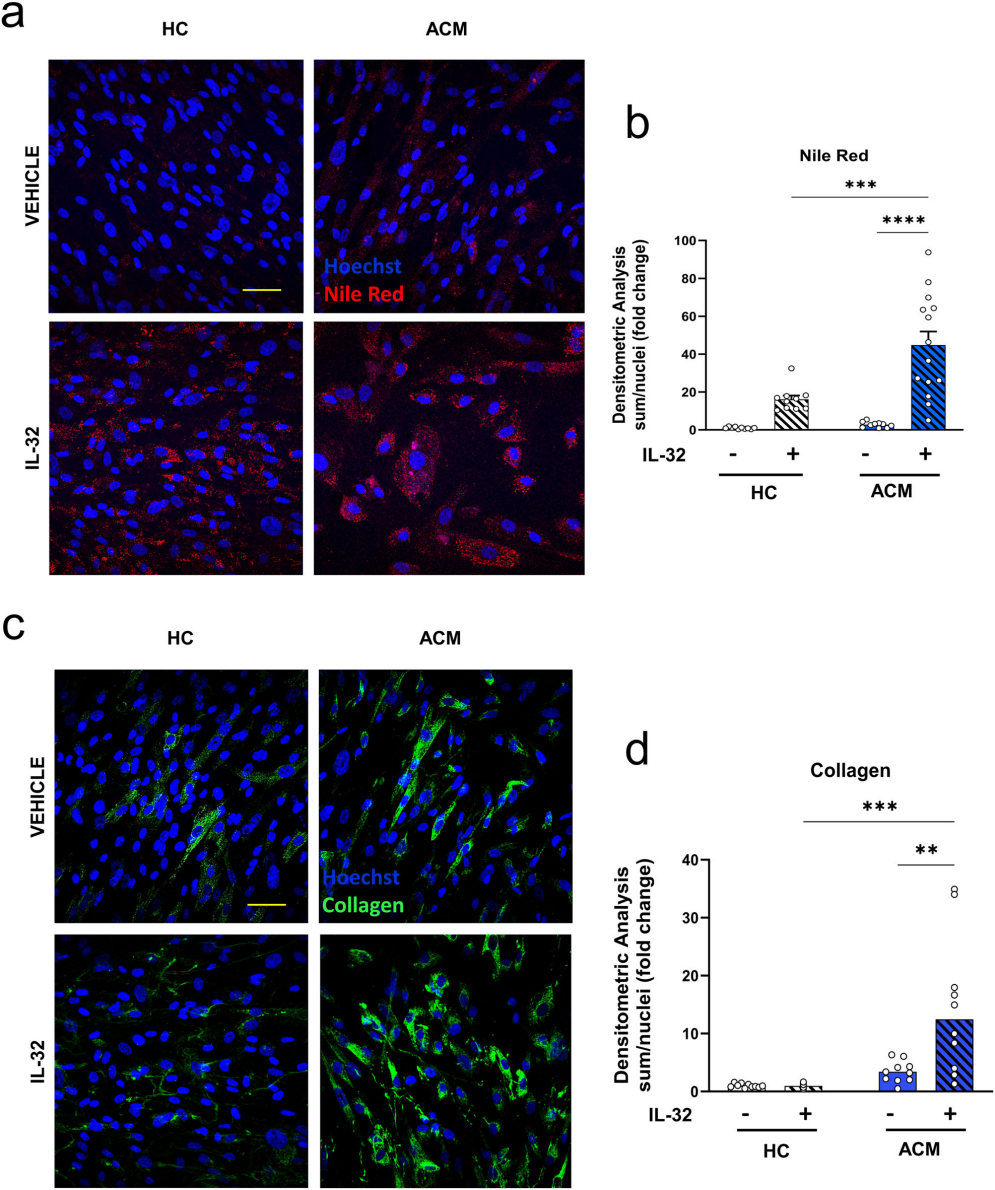
Adipogenic and pro-fibrotic effects of IL-32 on cardiac mesenchymal stromal cells (C-MSC) of ACM or HC. **a**, **b** Nile red staining showing lipid accumulation in C-MSC in basal conditions and after treatment with IL-32 20 ng/ml for 3 days (**a**), and its quantification (**b**) showed by sum nile red intensity per number of nuclei. **c**, **d** Immunohistochemistry showing collagen accumulation in C-MSC in basal conditions or after treatment with IL-32 20 ng/ml for 3 days (**c**) and its quantification (**d**) showed by sum 488 intensity per number of nuclei. Data are shown as mean ± SEM. ***p < 0.001, **p < 0.01, *p < 0.05 using two-way ANOVA test followed by Tukey’s multiple comparisons test. Scale bars: 50 µm.

Fibrofatty replacement is one of the pathological characteristics of ACM. In order to explore the possible effect of the secreted form of IL-32 (IL-32γ) on ACM pathogenesis, we treated C-MSC obtained from ventricular biopsy of ACM patients or HC with recombinant IL-32γ and evaluated lipid accumulation and collagen deposition. As previously reported, ACM C-MSC accumulate a similar amount of lipids as HC cells in growth conditions [4]. However, treatment with 20 ng/mL IL-32γ for 3 days significantly increased lipid accumulation, mainly in ACM C-MSC (Fig. 6a, b).

Collagen deposition was similar in ACM and HC C-MSC in basal conditions. Whereas control C-MSC did not respond to IL-32γ stimulation, 20 ng/mL of IL-32γ treatment for 3 days doubled collagen deposition in ACM C-MSC (Fig. 6c, d).

In contrast, treatment of CM derived from an ACM patient’s induced pluripotent stem cells (iPSC) [62] with 20 ng/mL IL-32γ showed no significant effect on contractility parameters (Fig. S8).

## Discussion

Our study demonstrates for the first time that ACM patients exhibit modified subpopulations of circulating immune cells with respect to healthy controls, mainly characterized by an increase of mTreg. Interestingly, mTreg subpopulations were characterized by an immunosuppressive phenotype that restrains immune response in ACM and supports tissue repair. Additionally, we identified IL-32 as highly expressed by blood tissue-like Treg subpopulation. It contributes to accelerating cardiac remodeling and dysfunction in ACM patients through lipid deposition and collagen accumulation and may constitute a promising target for the treatment of ACM fibrofatty pathogenesis.

Our data reveal that ACM patients exhibit altered PBMC subpopulations, notably affecting mTreg lymphocytes. Indeed, we observed a reduction in EMRA CD8^+^ T cells, along with an increase in naïve CD8^+^ T cells, suggesting impaired activation due to ACM-specific signals. In addition, classical monocytes showed significantly lower CD86 and HLA-DR expression in ACM, indicating an anergic phenotype with reduced antigen presentation. These results suggest that chronic low-grade inflammation in ACM [57, 63, 64] may hinder the conversion of naïve CD8^+^ T cells into memory cells. Furthermore, the lower abundance of Th1-like CD4^+^ T cells, NK cells, and EMRA CD8^+^ T cells, together with weakened monocyte antigen presentation, suggests a compensatory suppression of type 1 immune responses, known to be detrimental in HF [65]. The HLA-DR^lo/neg^ monocytes were shown to regulate the transition from the inflammatory state to immune suppression, ultimately leading to immune paralysis in patients with sepsis [39].

In ACM patients, the percentage of mTreg were significantly increased with mTreg expressing higher FOXP3 compared to controls. Increased FOXP3 expression typically enhances the suppressive function of Treg, making them more effective at controlling immune responses. This process is regulated by IL-6 in an inflammatory context, as described for mTreg from severe COVID-19 patients [50].

Treg master role is the down-modulation of immune responses and inflammation [66]. By controlling the activity of effector T cells and other immune cells, Treg can reduce tissue damage and inflammation [67, 68] [69]. In addition, they play a crucial role in tissue homeostasis, repair, and regeneration [70, 71]. In post-ischemic cardiac tissue, they exert their functions through the secretion of a variety of cytokines and growth factors, suppression of T-helper-dependent responses, and modulation of tissue macrophage functions [72]. Among them, anti-inflammatory cytokines and growth factors like IL-10, TGF-β, and Amphiregulin, help to reduce inflammation and promote tissue repair [73]. In ACM hearts, it is likely that a repair process is ongoing, to cope with CM death and stretch-induced damage [74]. Accordingly, excess TGF-β is described in ACM patients’ plasma and hearts [5, 75], and is prompting excessive extracellular matrix deposition.

Treg are also crucial for maintaining immune tolerance. Indeed, they help to inhibit autoimmune responses by suppressing CD4^+^ helper T cell subsets, such as Th1 or follicular helper T cells, controlling cell-mediated immunity and humoral responses. In ACM patients, both anti-DSG2 (a protein of the desmosome) and anti-heart autoantibodies have been described [22, 23]. Autoimmunity combined with genetically determined damage could trigger an excessive immune response, which Treg try to blunt.

Interestingly, in contrast to what happens in ACM, the frequency of Treg is underrepresented in dilated cardiomyopathy. Similarly, in hypertrophic cardiomyopathy hearts, the pathways linked to Treg activation are less expressed compared to controls [35, 76]. This may imply genetic-specific regulation of the immune response.

The single-cell transcriptomic profile of mTreg in ACM showed increased activation status, mediated by cytokines (TGFß, TNF-α, IFN-γ, IL-12, IL-6) or by TCR activation, suggesting that both antigen-dependent and independent responses could reinforce their suppressive activity. Interestingly, IL-6 has been shown to upregulate FOXP3 expression [50] and to strengthen repair capacities of muscle Treg for optimal adaptation to exercise and muscle regeneration after injury [54].

Effector Treg from cluster 5, with higher FOXP3 expression, were evocative of tissue-resident-like Treg [43], known to recirculate between blood and tissues under homeostatic conditions [56], exerting specific repair functions upon localization in injured tissues [72].

In ACM patients, mTreg showed an increased expression pattern of genes involved in Th1 but not Th17 suppression, in accordance with the lower frequencies of blood NK and EMRA CD8^+^T cells compared to HC. Furthermore, tissue localization and activation of ACM mTreg, suggested by the high expression of CXCR4-signaling, RhoA, N-cadherin, and integrin pathways, could be mediated by factors secreted from fibroblasts or myeloid cells of the ACM heart, such as osteopontin or TNF-α [57, 61].

In addition to their immunosuppressive activity, ACM Treg could participate in cardiac remodeling through high *IL-32* expression.

The IL-32 cytokine, induced by TNF-α [77], is known as a proinflammatory mediator [78], sustaining IL-6 and TNF-α signaling pathways and displaying higher gene expression levels in mTreg over other PBMC subsets [79]. In the context of inflammatory diseases, mTreg upregulate *IL-32* such as for patients with severe COVID-19 [50]. Furthermore, *IL-32* is expressed by PBMC of patients with heart failure and seric IL-32 concentrations have been positively correlated with cardiac fibrosis and poor outcomes after myocardial infarction [80], strongly suggesting its contribution to cardiac remodeling, through NFκB activation.

However, its expression was also associated with infiltration of Treg in a cancer model [81] suggesting that it could regulate Treg function through Foxp3 induction [82, 83].

IL-32 can be considered a unique cytokine since its structure does not match with most of the known cytokines and is expressed in different human tissues but is absent in mice [84, 85].

The IL-32γ is the complete and most active transcript within the six isoforms produced by alternative splicing such as the shorter and less harmful isoforms IL-32β or IL-32α [86–89].

Recent published scRNA-Seq datasets available on the CZ CELLxGENE Discover platform of non-cardiomyocyte cells from ACM hearts [61] allowed the identification of rare mem CD4^+^ T cells expressing *CD69*, *PRDM1,* and *IL-32*, comforting the accuracy of blood tissue-like mTreg migration in the pathogenic heart.

Recent reports highlighted the importance of cell-cell communication in ACM pathogenesis [62, 90]. Interestingly, immune-cell-derived IL-32 induced lipid accumulation as well as collagen deposition in C-MSC of ACM patients, even in the absence of stimulating media, revealing a triggering pathogenic action on heart tissue. Accordingly, IL-32 has been previously described as a modulator of the differentiation process of mesenchymal stem cells [91]. In addition, transgenic mice overexpressing IL-32 isoforms were characterized by increased pulmonary fibrosis [92] or greater accumulation of abdominal white adipose tissue [84], confirming the identified role of the cytokine in promoting fibro-adipogenesis.

Novel therapeutic approaches are envisioned for myocardial inflammation by the use of anti-inflammatory and immunomodulating therapy [93–95]. These results are encouraging, since both the arrhythmic and mechanical manifestations of the disease resulted improved. Some therapies, in particular, are targeting Treg to promote cardioprotective functions and regulate immune balance, cardiac and vascular remodeling, mediating immune tolerance, and promoting cardiac regeneration [96–98]. In the ACM context, the increased susceptibility of ACM patients to myocarditis [8, 64], which could be related to the Th1 suppressive tone exerted by ACM Treg, supports the development of therapeutic strategies targeting Th1-like Treg and the over-reparative phenotype of Treg.

Overall, the present study constitutes the first step to better understand the precise mechanisms by which effector Treg subsets influence ACM pathogenesis. These findings are essential to develop therapies that can enhance Treg protective functions while repressing side deleterious effects. In this line, IL-32 represents a promising target for the treatment of ACM fibrofatty pathogenesis.

## Material and methods

### Ethical statement

This study complies with the declaration of Helsinki and was approved by the Centro Cardiologico Monzino Ethics Committee (R1020/19-CCM1072; date of approval: 3/7/2019). Written consent was signed by all participating subjects.

### Patient recruitment and sample collection

Thirty-six ACM patients were recruited in the Centro Cardiologico Monzino (Milan, Italy). Samples' use for research was approved by Centro Cardiologico Monzino and Istituto Europeo di Oncologia ethics committee (R1020/19-CCM1072; date of approval: 3/7/2019). Written consent was signed by the participating subjects. The ACM diagnosis was confirmed via assessment of clinical, imaging, anatomo-pathological, and genetic results (Table S1).

Right ventricle samples were obtained from ACM patients undergoing catheter biopsies (as previously described [99]).

Venous blood samples (about 20 ml) from patients were collected on sodium heparin tubes (BD vacutainer) from ACM patients during routine laboratory tests and from age- and sex-matched healthy donors who tested negative for HIV, HBC, HCV from EFS (Etablissement Français du Sang Toulouse).

### PBMC isolation

PBMC were isolated by Ficoll Paque density gradient separation. Briefly, 20 ml of peripheral blood used for this protocol was diluted with 20 ml of sterile Phosphate-Buffered Saline (PBS). The diluted blood was then gently transferred and layered to two sterile tubes containing 15 ml of Ficoll (GE Healthcare). The samples were then centrifuged 2500 rpm (1306 g) for 15 min at room temperature in a swinging-bucket rotor without brake. The upper layer containing plasma was collected and frozen while the mononuclear cell layer was collected and transferred into a new sterile tube. Cells were washed twice by adding sterile PBS and centrifuged at 1800 rpm (677 g) for 10 min at 4 °C. After removing the supernatant, the cell pellet was resuspended in Cryostor CS10 cell preservation media (Stem Cell Technologies) and cryopreserved.

### Primary cardiac stromal cell culture and treatment

C-MSC were isolated from ventricular samples and cultured onto uncoated Petri dishes (Corning) as previously reported [4, 99]. The cells were cultured in a growth medium consisting of IMDM supplemented with 20% fetal bovine serum (FBS; Euroclone), 10 ng/mL basic fibroblast growth factor (R&D Systems), 10,000 U/mL penicillin (Invitrogen), 10,000 µg/mL streptomycin (Invitrogen), and 20 mmol/L L-Glutamine (Sigma–Aldrich). The treatments were performed in a culture medium and specifically by adding 20 ng/mL of IL-32 (R&D Systems) for 3 days. The concentration has been chosen after a literature search [100, 101] and a dose-response curve (Fig. S9).

### Differentiation of iPSC into CM

iPSC-CM cells were generated from undifferentiated hiPSC through the induction cardiac mesoderm as previously described [62, 102]. In brief, 25,000 cells per cm^2^ were seeded on Matrigel on day -1. On day 0, cardiac mesoderm was induced by Low insulin - BSA based - Polyvinyl Alcohol - Essential Lipids (LI-BPel) medium (composition: IMDM, F12, Protein Free Hybridoma Medium-II, BSA, PVA, Chemically Defined Lipid Concentrate, Insulin-Transferrin-Selenium-Ethanolamine, α-Monothioglycerol, L-ascorbic acid 2-phosphate, Glutamax, Penicillin-streptomycin, Phenol red [102], supplemented with a mixture of cytokines (20 ng/mL BMP4, R&D Systems; 20 ng/mL ACTIVIN A, Miltenyi Biotec; 1.5 μM GSK3 inhibitor CHIR99021, Selleckchem)). After 3 days, cytokines were removed and the WNT inhibitor XAV939 (5 μM, Tocris) was added for 3 days. On day 6, the medium was replaced with LI-BPel medium without supplement. The medium was refreshed every 2–3 days until the beating cells appeared. Metabolic selection of iPSC-CM with 4 mM sodium-L-lactate was performed to maximize CM enrichment. After the selection with lactate medium was replaced with LI-BPel.

### Flow cytometry and cell sorting

Phenotypic analysis of PBMC was performed using multicolor flow cytometry. Briefly, non-specific binding of antibodies by Fc receptors was blocked by incubating the cells with TruStain FcX™ or True-Stain Monocyte Blocker™ from Biolegend diluted in FACS buffer (PBS 4% heat-inactivated FBS and 2 mM EDTA). After centrifugation, cells were labeled with fluorochrome-conjugated antibodies to assess the frequency of lymphocyte subsets and monocytes, memory T cell subsets, or monocyte activation markers (Table S2). Viability was assessed by incubation with Live-Dead Yellow (Invitrogen). The acquisition was performed on an LSR Fortessa (BD Bioscience) at the Flow Cytometry and Cell Sorter facility of the I2MC Institute. Analysis of flow cytometric data was performed using FlowJo (TreeStar).

Memory CD45RO^+^ CCR4^+^ CD25^+^ CD4^+^ T cells were sorted from PBMC healthy or ACM donors by staining with anti-CD4, CD3ε, CD45RO, CD25, CCR4, CCR7, CD14, CD19, and CD56. Identification of ACM or HC cell donors was performed by barcoding with TotalSeg anti-human Hashtags antibody (respectively for each donor, A025, A0252, A0253, A0254 or A0255; Biolegend) and then sorted on an INFLUX cell sorter (BD Biosciences). Cell fractions were collected in X-Vivo15 (Lonza) supplemented with 2% heat-inactivated human AB serum (Sigma–Aldrich), pooled per condition, and counted by trypan blue exclusion with 44,000 cells for ACM patients and 23,100 for controls.

### Single-cell RNA sequencing

12000 sorted memory CD45RO^+^ CCR4^+^ CD25^+^ CD4^+^ T cells from ACM patients or controls (Table S3) were injected and with a capture rate of 40%, 5000 cells were partitioned into nanoliter-scale Gel Bead-In-Emulsions (GEMs) using the Single-Cell 3′ reagent kits v2 (Chromium 10x Genomics). Barcoded cDNAs were then amplified by PCR to generate sufficient mass for library construction. Sequencing was performed using the Illumina Nova-Seq 6000 system (Illumina) in an average sequencing read depth of 304 K reads/cell.

#### Pre-processing

Barcode ranks plots: Droplets with less than 100 counts were used to make up the ambient RNA pool, droplets having profiles significantly different from the ambient RNA were considered to contain cells.

#### Quality control

In this step, the dataset was explored and filtered to remove low-quality cells and lowly or highly expressed genes. Cells were then filtered using MADs for (log10) and Minimum threshold for (log10) parameters. Low-abundance genes were defined as those with an average count below a filter threshold of −2.69 (−0.5 MADs). High-abundance genes had an average count above a filter threshold of 2.34 (3 MADs). The multi–Batch Norm function was used for normalization. Doublets captured in the same droplets are considered low-quality cells. Doublet detection strategy was performed using the Compute Double Density function from the scDblFinder package. Data integration was performed using the Correct Experiments function in the batchelor package. Single-cell raw data are deposited in the GEO repository with the record number GSE262428.

### Quantitative real-time PCR

Total RNAs were purified using the ReliaPrep RNA Cell Miniprep System (Promega) according to the manufacturer’s instructions. RNA was quantified with NanoDrop (ND 2000, Thermofisher). RNAs were retrotranscribed with the SuperScript VILO cDNA Synthesis Kit (ThermoFisher Scientific). Real-time qPCR reactions were performed using TB Green Premix Ex Taq II (Takara) on a ViiA7 Real-Time PCR System (Applied Biosystems) instrument using a specific primer pair (Table S4). Gene expression levels were normalized to GAPDH and 36B4 housekeeping genes. Results were analyzed using the ΔCT method.

### Immunofluorescence on tissues and cells

To perform an immunofluorescence assay, paraffin was removed from embedded RV endomyocardial bioptic sections from ACM patients and control samples. Antigen unmasking was performed by heating sections at 90 °C in antigen retrieval buffer pH 6 (DAKO, Santa Clara, USA). For cell immunofluorescence, C-MSC were fixed using 4% paraformaldehyde in PBS.

After blocking with PBS supplemented with 5% BSA and 0.1% Triton X-100 (PBS-T/BSA) for 60 min, the slides were incubated with specific primary antibodies (as reported in Table S5) overnight (O/N) at 4 °C. Fluorescence-labeled secondary antibodies (Invitrogen, Carlsbad, CA, USA) were added for 1 h at RT. Otherwise, fixed cells were stained using 12.5 ng/ml of Nile Red (Invitrogen, Carlsbad, California, USA) for 1 h at RT. Nuclei were stained with Hoechst 33342 (Sigma−Aldrich, Saint Louis, MO, USA). Images were acquired with a confocal microscope in Z-stack mode with a 40× oil immersion objective (Zeiss LSM710-ConfoCor3 LSM, Zeiss, Germany) using the software Zen 2008 (Zeiss, Germany). Fluorescence signal quantification was performed using ImageJ software on Z-Stacks images. Single channels from each image were converted into 8-bit grayscale images and thresholded in order to subtract the background. The fluorescence value has been normalized to the number of nuclei per field (at least 5 for each experiment). Nuclei counting was performed using the ImageJ tool.

### IL-32 quantification in human plasma samples

IL-32 serum levels of ACM patients (Table S1) and sex/age-matched controls were quantified using the Human IL-32 DuoSet ELISA kit (R&D systems) following the manufacturer’s instruction.

### Statistical analysis

Continuous variables are presented as mean ± standard error (SEM), and categorical data as counts and proportions. A normality test was performed for each sample variable. Normally distributed continuous variables were compared using Student’s *t*-test for independent samples. Age- and sex-matched samples were compared using the Wilcoxon matched-pairs signed rank test. Unpaired groups were analyzed using the unpaired *t*-test or Mann–Whitney *t*-test. Comparisons among three or more groups were performed with one-way or two-way ANOVA test, in association with Tukey’s or Dunnett’s multiple comparison post-hoc tests. A *p*-value < 0.05 was considered statistically significant except where indicated. The statistical evaluation of outliers was performed before exclusion. Statistical analyses and graphics were done GraphPad Prism 9.

## Supplementary information

Supplementary text and figures

## Data Availability

The data supporting the findings of this study are available within the article and its Supplementary Materials. Additional supporting data are available from the corresponding author upon reasonable request. The single-cell raw data are available in the GEO repository (ID: GSE262428).
