# Supplementary material for: Analysis of effector/memory regulatory T cells from arrhythmogenic cardiomyopathy patients identified IL-32 as a novel player in ACM pathogenesis
Source: Cell Death Dis. 2025 Feb 11;16(1):87. doi: 10.1038/s41419-025-07364-y (PMC11814135; doi:10.1038/s41419-025-07364-y)
Supplement: Supplementary file 1 — Supplementary text and figures [file 41419_2025_7364_MOESM1_ESM.pdf]

**Title**

Analysis of effector/memory regulatory T cells from Arrhythmogenic Cardiomyopathy patients identified IL-32 as a novel player in ACM pathogenesis

**Running title:** Treg in ACM

Authors

Salwa Soussi*, Angela Serena Maione*, Lise Lefèvre, Nathalie Pizzinat, Jason Iacovoni, Ignacio Gonzalez-Fuentes, Daniel Cussac, Lara Iengo, Yohan Santin, Fabrizio Tundo, Claudio Tondo, Giulio Pompilio, Angelo Parini£, Victorine Douin-Echinard# and Elena Sommariva#.

* Contributed equally as first authors

# Contributed equally as last authors

£ Corresponding author: Angelo Parini, I2MC, INSERM, UMR-1297, Toulouse, France, Email: angelo.parini@inserm.fr

## Supplementary text

Using published Treg gene signatures (1–5), we generated a bi-clustering heatmap of the gene-gene correlation matrix of ACM and HC CCR4$^{+}$ CD25$^{+}$ CD45RO$^{+}$ T cells (**Figure S5**), revealing gene modules associated with tissue residency (*KLF6, PRDM1, CXCR4, CD69, PTGER4, NR4A2*), tissue repair (*AREG, IRF1, TNF, CITED2; GPX4, TIMP1, ANXA1, FLT3LG, VEGFB, MYDGF*) (6), cell activation (*IL-32, LGALS1, S100A4, S100A9, CD74, HLA-DR*) and effector functions *(NEAT1, NR3C1, CTLA4, CTS7*), and central memory (*PTPRC, S1PR4, SELL, CD27, LY6E, CORO1A*) (**Figure S5**). The bi-clustering heatmap also showed correlation between canonical signature genes of Treg activation (*FOXP3, TNFRS1B, IL-2RA, ICOS, CD2*), proliferation (*TUBB, CD59, MKI67, LIMS1*) (1) and effector functions (*AHR, IRF4, SEMA4D, VSIR, STAT1*) (**Figure S5**). Most of the gene expression modules overlapped the six clusters, but higher gene expression of tissue residency module was observed for cluster 5, of repair module for cluster 1, and of activation module for cluster 6. Cluster 4 was characterized instead by the lowest expression of tissue residency genes and high level of central memory genes (**Figure S5**).

## Supplementary figures

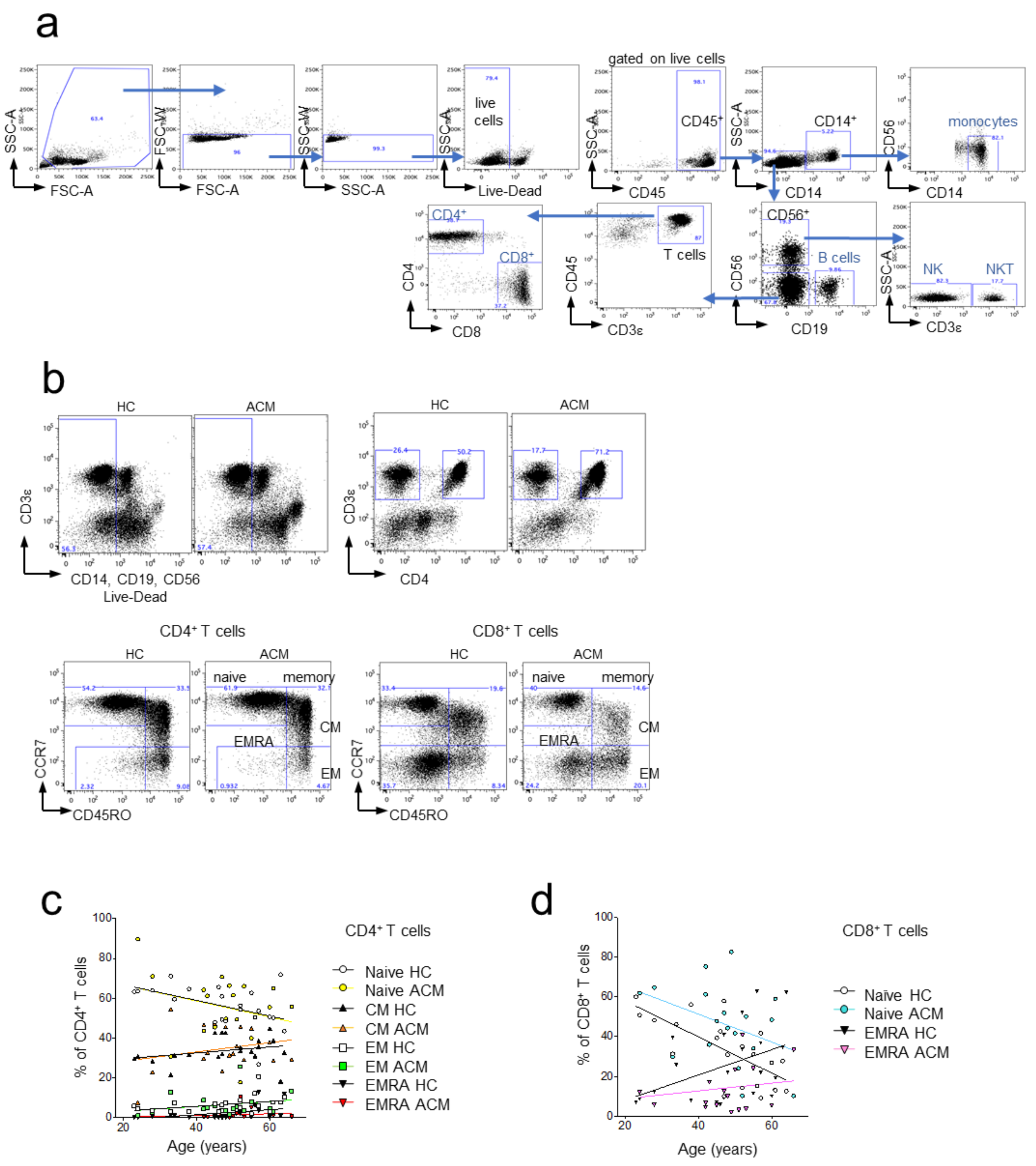

**Figure S1. Gating strategy defining the immune cells subsets and memory T lymphocyte subsets in PBMC of HC and ACM patients.**

(**a**) Live cells were gated based on negative Live-Dead staining. Gated $CD45^+$ single cells were plotted for CD14 expression and monocytes were gated in the $CD56^-$ $CD14^+$ fraction. The B lymphocytes were selected based on negative expression of CD14 and CD56 and positive expression of CD19. NK and NTK were gated using CD56 positive staining and differentiated with the expression of CD3ε. The $CD45^+$ CD3ε+ T lymphocytes were further discriminated by population gating on the $CD4^+$ and $CD8^+$. (**b**) Dot plots showing an example of the gating strategy excluding dead cells, $CD14^+$, $CD19^+$ and $CD56^+$ cells, to study memory T lymphocyte subsets from one HC and one ACM patient based on CCR7 and CD45RO staining. (**c, d**) Age-related changes of the percentage of naive and memory cell subsets in $CD4^+$ (c) and $CD8^+$ T (d) T cells of HC and ACM patients.

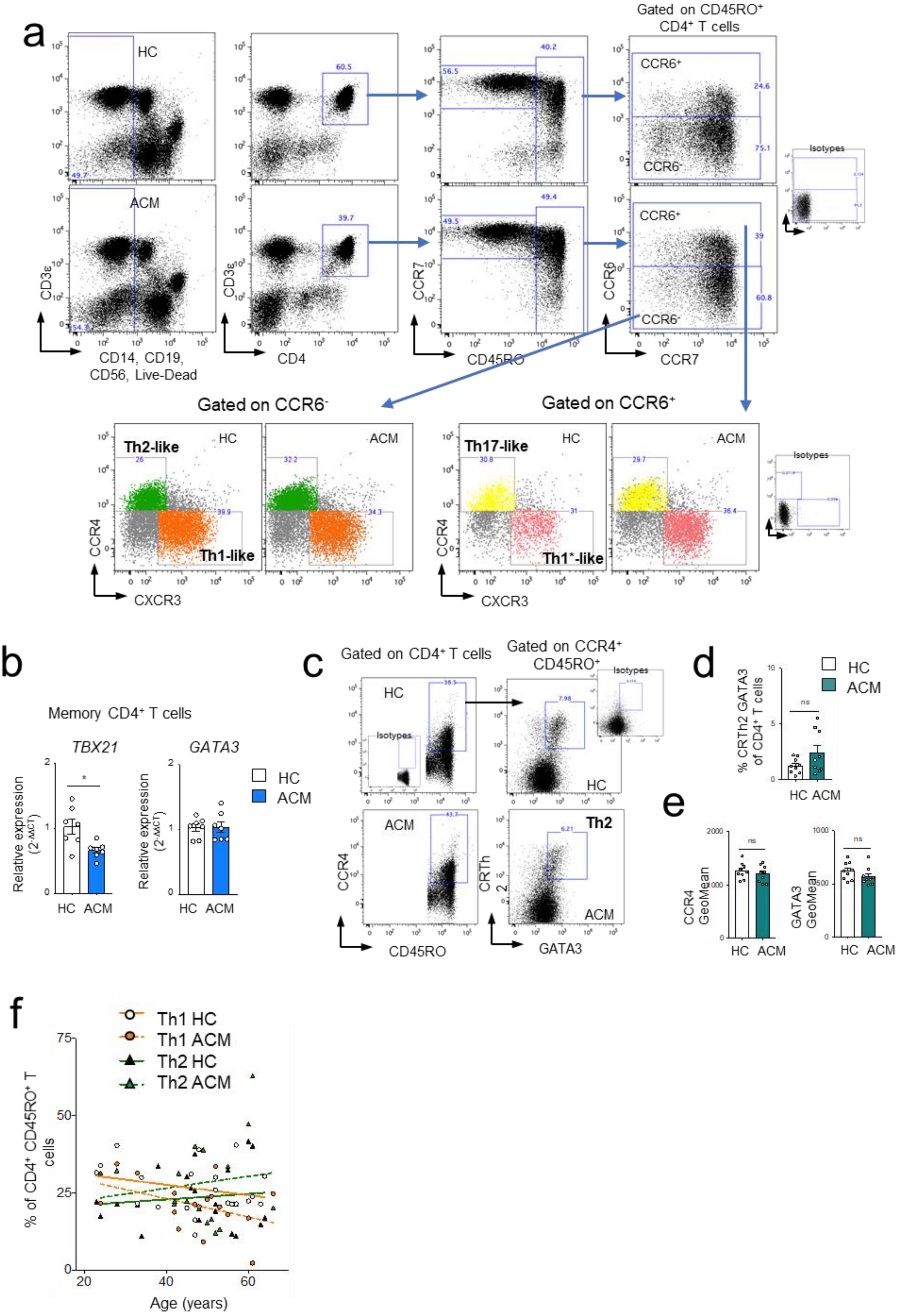
a
HC
ACM
CD3ε
CD14, CD19, CD56, Live-Dead
CD4
CCR7
CD45RO
Gated on CD45RO+ CD4+ T cells
CCR6+
CCR6-
CCR6
Isotypes
Gated on CCR6-
Gated on CCR6+
Th2-like
Th1-like
Th17-like
Th1*-like
CCR4
CXCR3
b
Memory CD4+ T cells
TBX21
GATA3
Relative expression (2-ΔΔCT)
HC
ACM
c
Gated on CD4+ T cells
Gated on CCR4+ CD45RO+
CRTh2
GATA3
Th2
d
% CRTh2 GATA3 of CD4+ T cells
ns
e
CCR4 GeoMean
GATA3 GeoMean
f
Th1 HC
Th1 ACM
Th2 HC
Th2 ACM
% of CD4+ CD45RO+ T cells
Age (years)

**Figure S2. Gating strategy and markers used to identify the CD4+ helper T subsets in PBMCs of HC and ACM patients.**

(**a**) Gating strategy to identify Th1-like, Th2-like, Th17-like and Th1*-like subsets of $CD4^+$ $CD45RO^+$ helper T cells based on chemokine receptor expression is shown for one representative HC and ACM patient. (**b**) Relative expression of TBX21 and GATA3 by memory $CD45RO^+$ $CD4^+$ T cells of ACM patients relative to HC control group (n=7 per group). (**c-e**) Example of gating strategy to identify Th2 cells using CRTh2 and GATA3 staining for one HC and one ACM patient (c) and percentages among $CD4^+$ $CD45RO^+$ T cells of HC and ACM (n=9 per group) are shown (d). Histograms showed geometric mean (e) of CCR4 and GATA3 for gated Th2 cells from HC and ACM patients. (a, c) Dot plots of isotype control staining for CCR6 and CCR4 (a), and for CCR4 and GATA3 (c) for baseline detection are shown. (**f**) Age-related changes of the percentage of Th1-like and Th2-like cell subsets in memory CD45RO $CD4^+$ T cells of HC and ACM patients are shown. Data are shown as mean +/- SEM. * $p<0.05$ and ns not significant using Mann Whitney test.

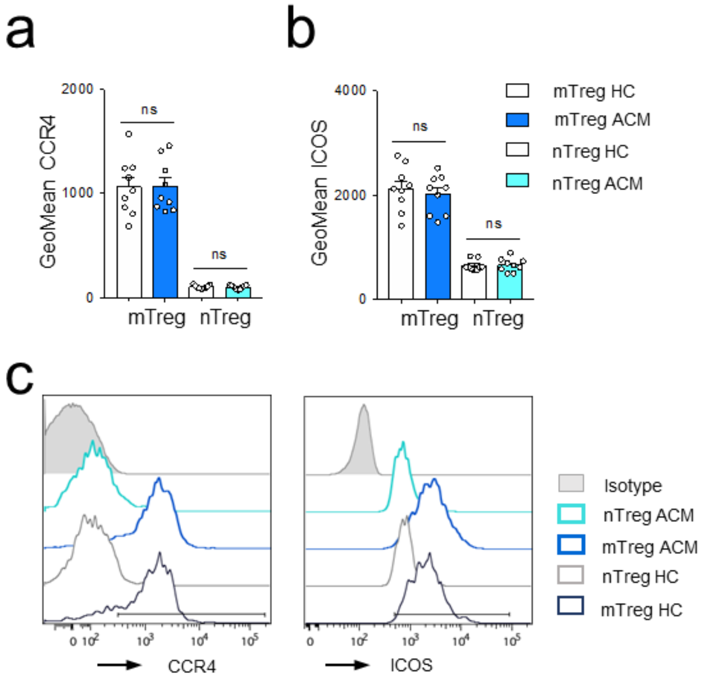


**Figure S3. Expression of CCR4 and ICOS markers by Treg subsets in HC and ACM patients.**

(**a, b**) Geometric fluorescence mean of CCR4 (a) and ICOS (b) for memory Treg (mTreg) and naive Treg (nTreg) from HC and ACM patients (n=9 per group). (**c**) Fluorescence histogram overlay showed examples of CCR4 and ICOS expression by naive ($CD45RO^-$) or memory ($CD45RO^+$) Treg from one HC and one ACM patient. Data are shown as mean +/- SEM. ns, not significant using Wilcoxon matched-pairs signed rank test.

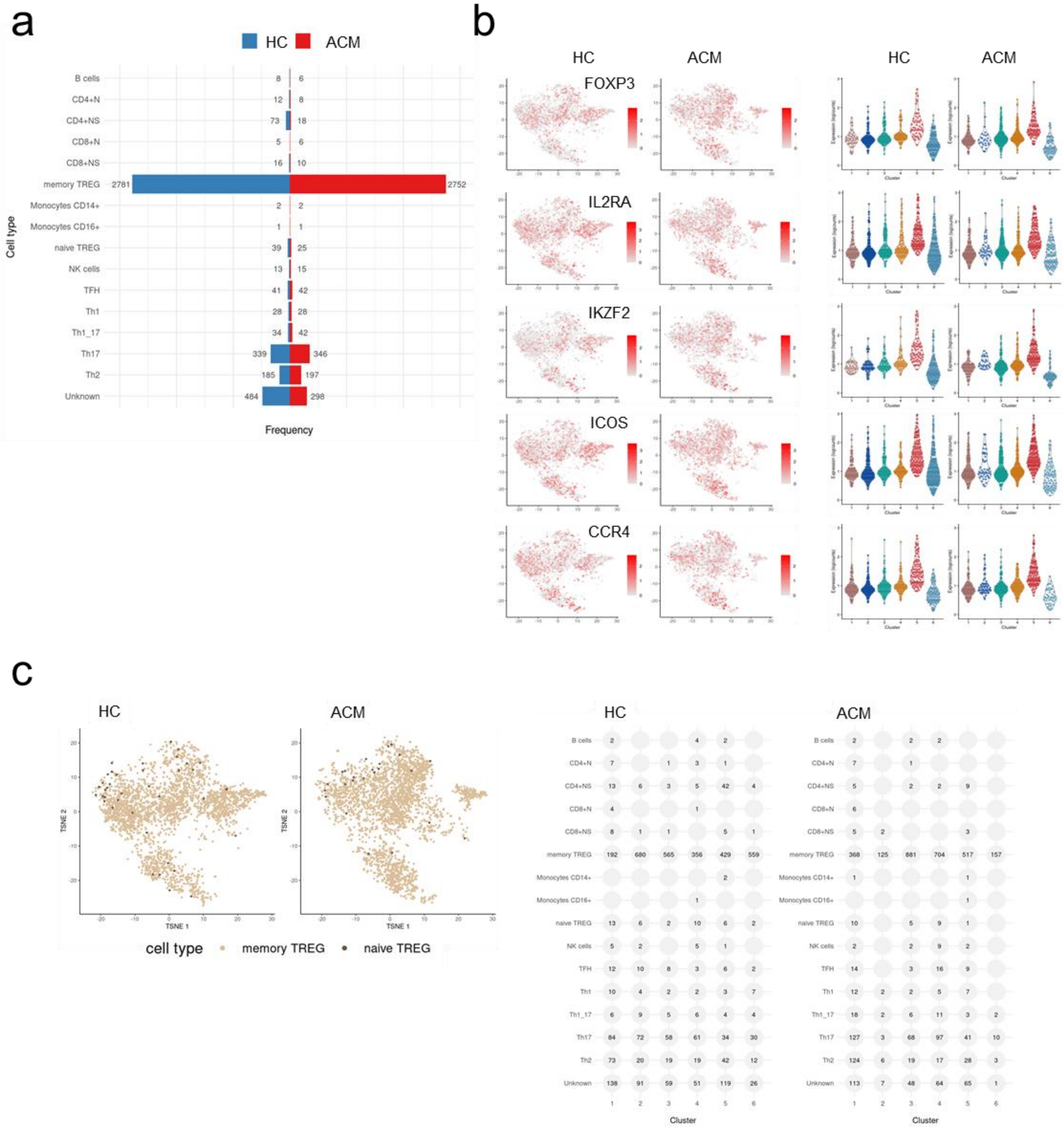
a
HC
ACM
B cells
CD4+N
CD4+NS
CD8+N
CD8+NS
memory TREG
Monocytes CD14+
Monocytes CD16+
naive TREG
NK cells
TFH
Th1
Th1_17
Th17
Th2
Unknown
Cell type
Frequency
8 6
12 8
73 18
5 6
16 10
2781 2752
2 2
1 1
39 25
13 15
41 42
28 28
34 42
339 346
185 197
484 298
b
HC
ACM
FOXP3
IL2RA
IKZF2
ICOS
CCR4
c
HC
ACM
TSNE 1
TSNE 2
cell type
memory TREG
naive TREG
Cluster

**Figure S4. ScRNA-Seq analysis of mTreg cells from HC and *PKP2*-mutated ACM patients.**

(**a**) Histograms showing the relative proportions and numbers of HC and ACM cells in the different immune cell populations identified in the scRNA-seq dataset using the DICE database (7). (**b**) Expression of *FOXP3*, *IL-2RA*, *IKZF2*, *ICOS* and *CCR4* by HC or ACM cells projected on the t-SNE plot and represented as violin plots for the respective clusters. (**c**) T-SNE projection showing mTreg and nTreg in the different clusters, thanks to annotation of human immune cell types using the DICE database and numeration of the different immune cell populations in the 6 clusters of ACM *PKP2*-mutated patients (*PKP2**) and of age- and sex-matched HC.

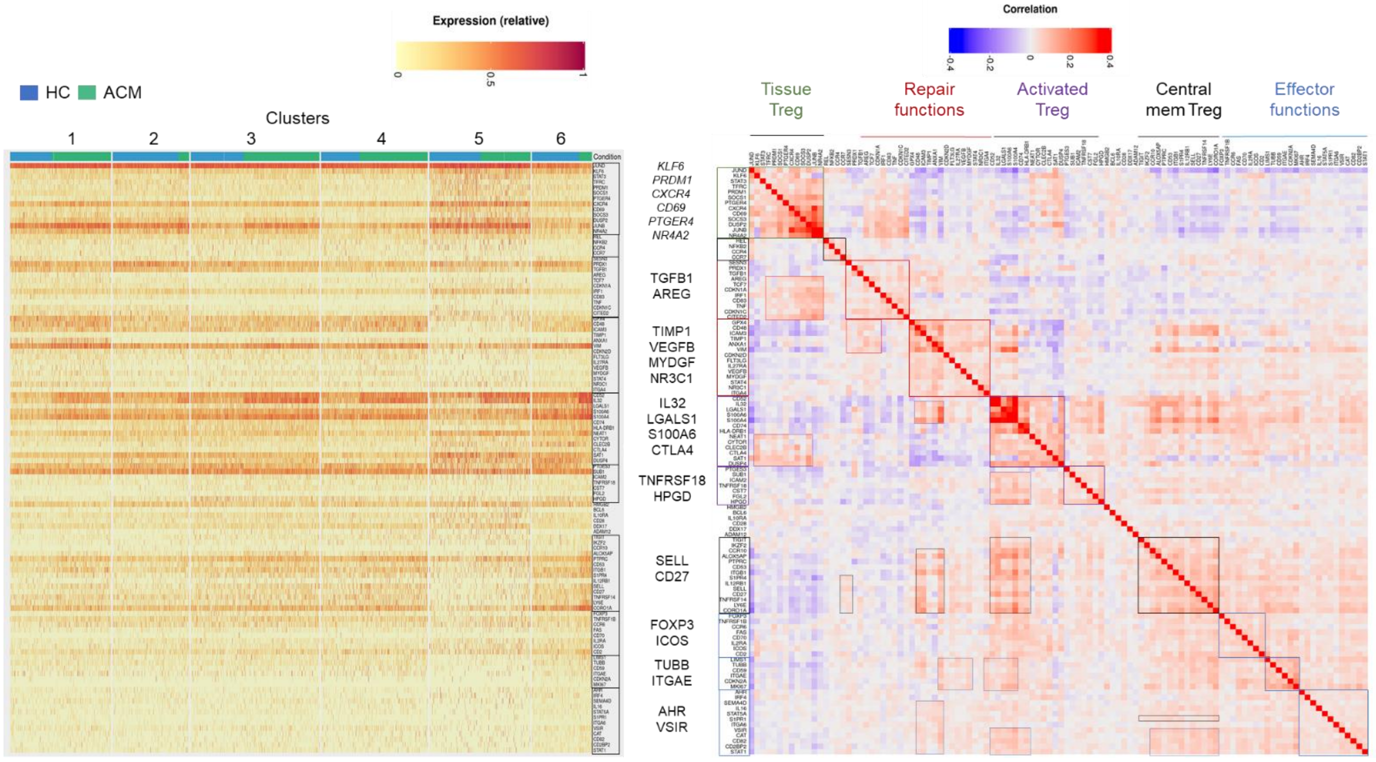


**Figure S5. Bi-clustering heatmap of gene-gene correlation matrix of ACM and HC mTreg.**

Clustering heatmap of the gene-gene correlation matrix of signature mTreg genes based on Spearman's rank correlation (right) and heatmap of the relative expression of these genes in the 6 clusters (left), each vertical line representing a cell.

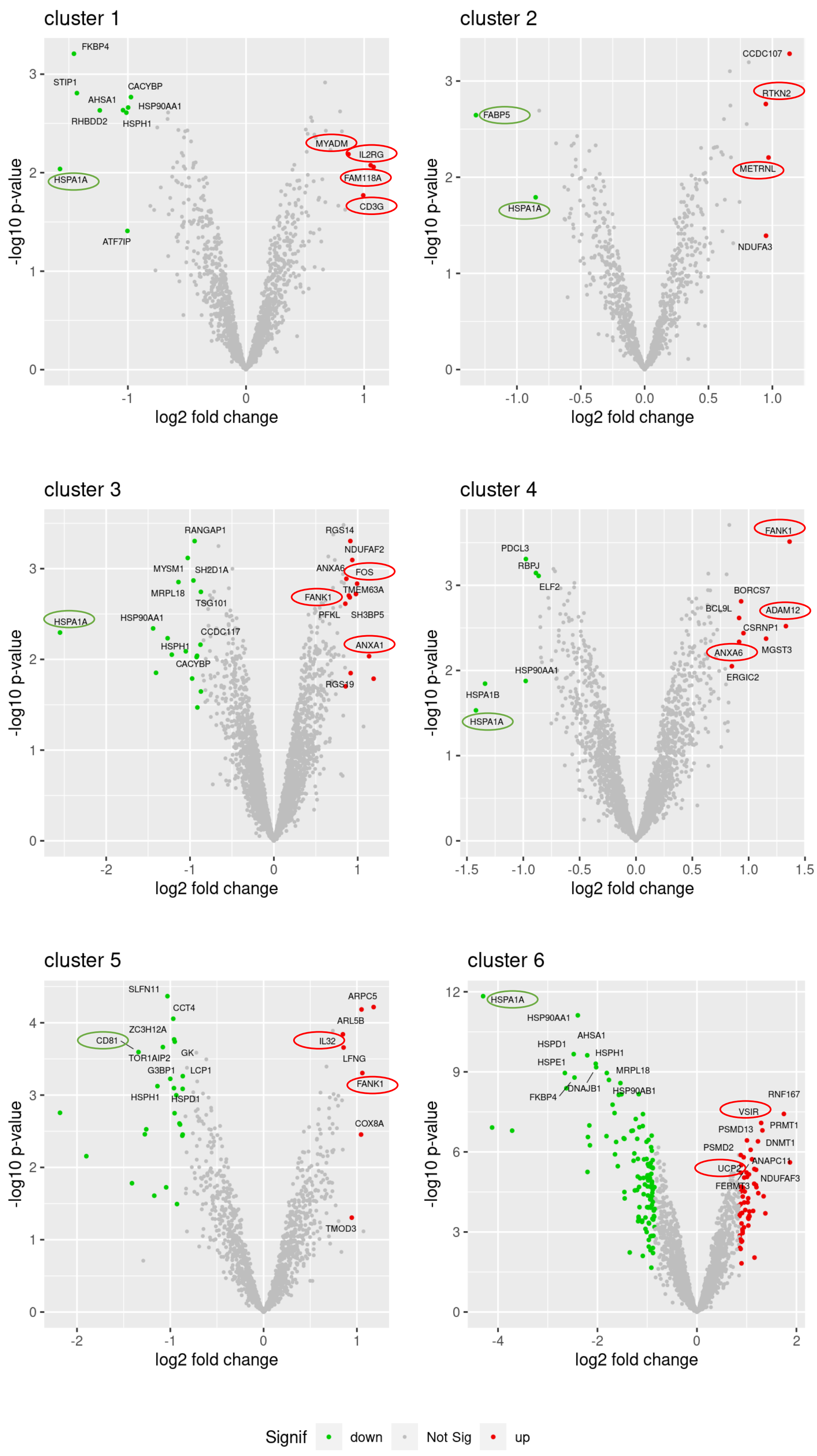

cluster 1
cluster 2
cluster 3
cluster 4
cluster 5
cluster 6
-log10 p-value
log2 fold change
FKBP4
STIP1
CACYBP
AHSA1
HSP90AA1
RHBDD2
HSPH1
HSPA1A
ATF7IP
MYADM
IL2RG
FAM118A
CD3G
CCDC107
RTKN2
FABP5
METRNL
NDUFA3
RANGAP1
RGS14
NDUFAF2
MYSM1
SH2D1A
ANXA6
FOS
MRPL18
TMEM63A
TSG101
FANK1
HSP90AA1
PFKL
SH3BP5
CCDC117
ANXA1
HSPH1
CACYBP
RGS19
PDCL3
RBPJ
ELF2
BORCS7
BCL9L
ADAM12
CSRNP1
MGST3
ERGIC2
HSPA1B
SLFN11
CCT4
ARPC5
ARL5B
ZC3H12A
CD81
IL32
TOR1AIP2
GK
LFNG
G3BP1
LCP1
HSPD1
COX8A
TMOD3
HSPE1
FKBP4
DNAJB1
HSP90AB1
RNF167
VSIR
PSMD13
PRMT1
PSMD2
DNMT1
UCP2
ANAPC11
NDUFAF3
FERMT3
Signif
down
Not Sig
up

**Figure S6. Differential gene expression analysis of ACM vs. HC cells**

Volcano plot representing significantly differentially expressed genes between ACM and HC cells within each identified cluster. Each point shows individual gene (|logFC| > 0.85 and P-Value < 0.05) which are colored by their $log_2$ fold change.

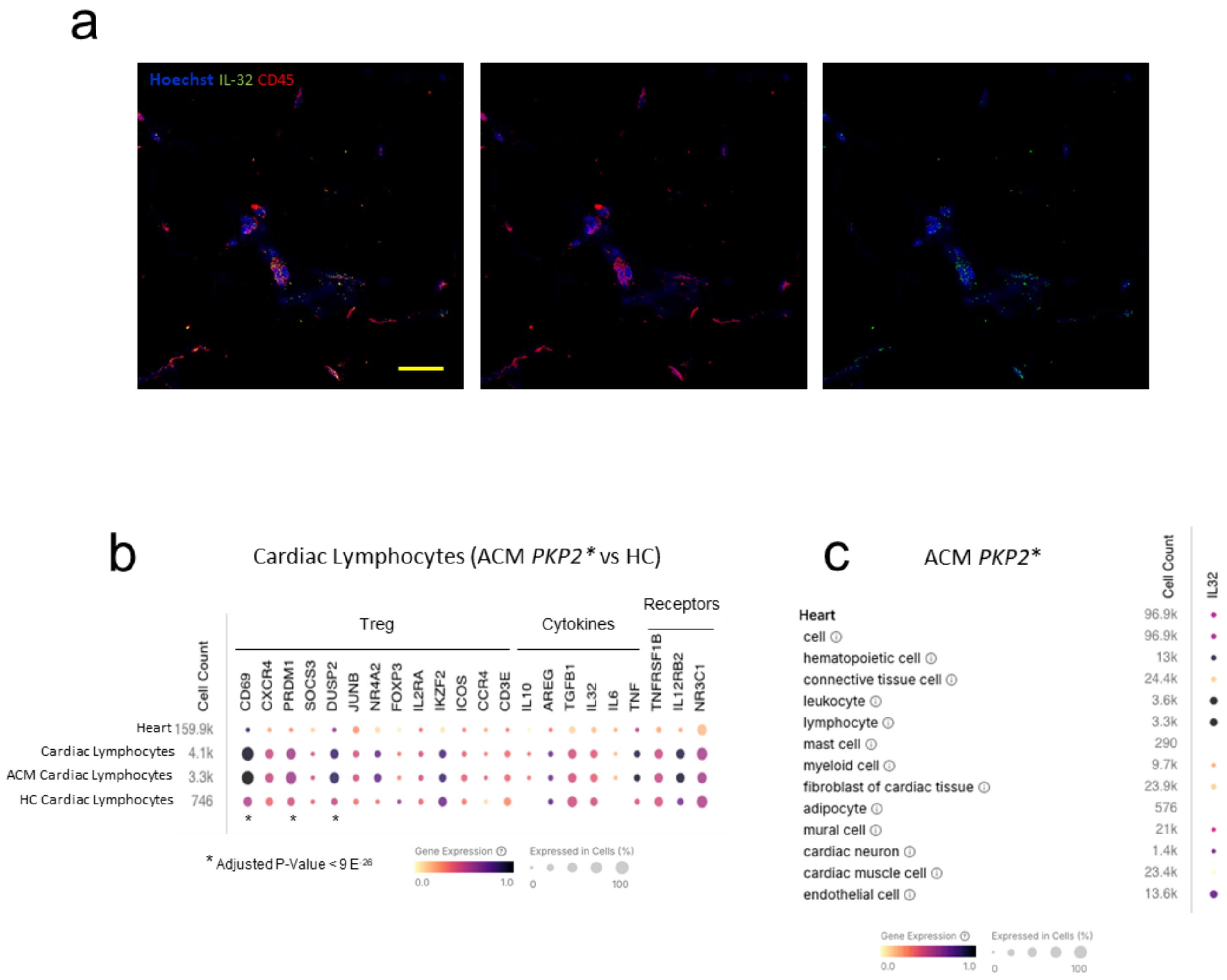


**Figure S7. IL-32 expression by CD45$^{+}$ hematopoietic cells and lymphocytes in cardiac tissue of ACM patients.**

(**a**) Immunofluorescence images of cardiac biopsies sections from an ACM patient stained with anti-IL-32 and anti-CD45 antibodies. The green laser was kept low to see only the highest signal for IL-32. Scale bars: 50 µm. (**b, c**) Analysis of *IL-32* gene expression by cardiac cells (b) and lymphocytes (c) from ACM *PKP2** patients and HC from scRNA-seq dataset published by Reichart et al. Science 2022 (8). Transcripts associated with tissue-residency markers, the mTreg core signature, as well as cytokines and receptors, are displayed using the CZ CELLxGENE platform (9).

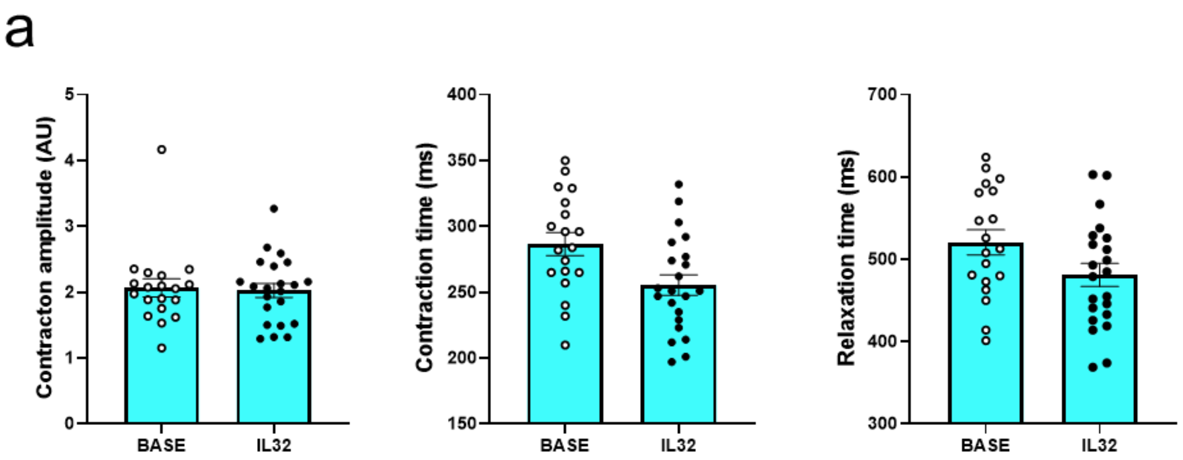


**Figure S8. ACM iPSC-derived CM treated with IL-32γ showed no significant effect on contractility parameters.**

iPSC-CM were treated with 100ng/mL of IL-32γ, and contraction kinetics including contraction amplitude, contraction time and relaxation time were measured at 37°C based on CytoMotion system during field stimulation at 1 Hz (n=19, BASE; n=22, IL-32; Two-tailed Student's t-tests).

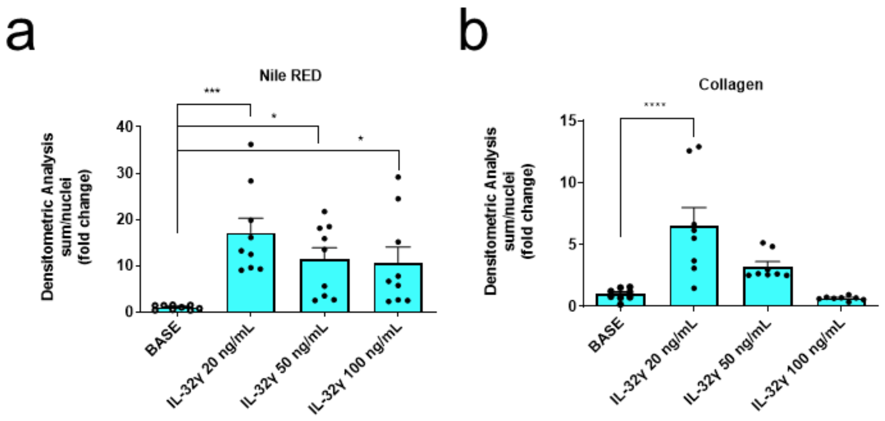


**Figure S9. IL-32 treatment dose response curve.**

Different doses (20, 50 or 100 ng/mL) of recombinant IL-32γ were used to treat ACM C-MSC and fluorescence for Nile Red staining (**a**) or collagen I expression (**b**) was evaluated compared to untreated (Base).

## Supplementary tables

**Table S1: Characteristics of patients who donated blood.**

M: male; F: female; ACM: Arrhythmogenic Cardiomyopathy; *PKP2**: carrier of pathogenic or likely pathogenic variant in the plakophilin gene.

| id | Age (Years) | Sex | Diagnosis |
|---|---|---|---|
| M003 | 42 | M | ACM *PKP2** |
| M020 | 41 | M | ACM *PKP2** |
| M031 | 58 | M | ACM |
| M035 | 39 | F | ACM *PKP2** |
| M056 | 51 | M | ACM |
| M061 | 41 | M | ACM *PKP2** |
| M062 | 31 | M | ACM |
| M064 | 46 | M | ACM |
| M068 | 65 | M | ACM |
| M069 | 59 | M | ACM |
| M070 | 24 | F | ACM |
| M071 | 20 | M | ACM |
| M074 | 52 | M | ACM |
| M075 | 49 | F | ACM |
| M077 | 41 | M | ACM *PKP2** |
| M079 | 49 | M | ACM |
| M080 | 52 | F | ACM |
| M082 | 49 | F | ACM |
| M083 | 52 | M | ACM |

| | | | |
|---|---|---|---|
| M108 | 38 | M | ACM |
| M111 | 49 | F | ACM |
| M115 | 28 | F | ACM |
| M117 | 43 | M | ACM |
| M118 | 24 | F | ACM |
| M124 | 34 | M | ACM |
| M137 | 59 | M | ACM |
| M072 | 61 | M | ACM |
| M081 | 18 | M | ACM |
| M097 | 39 | M | ACM |
| M104 | 24 | M | ACM |
| M106 | 33 | M | ACM |
| M112 | 66 | M | ACM |
| M116 | 34 | F | ACM *PKP2** |
| M120 | 32 | M | ACM |
| M121 | 65 | M | ACM |
| M127 | 53 | M | ACM |

**Table S2: List of antibodies used for flow cytometry**

| Fluorophore | Target | Clone | Provider |
|---|---|---|---|
| PercP-Vio700 | CD3ε | REA613 | Miltenyi |
| Viobright FITC | CD4 | REA623 | Miltenyi |
| PE | CD25 | BC96 | Biolegend |
| PE-Vio770 | CCR4 | REA279 | Miltenyi |
| AF647 | FOXP3 | 150D | Biolegend |
| APC-Vio770 | CD45RO | REA747 | Miltenyi |
| BV421 | ICOS | C398.A4 | Biolegend |
| BV421 | CCR7 | G043H7 | Biolegend |
| PE | GATA3 | 16E10A23 | Biolegend |
| AF647 | CRTh2 | BM16 | Biolegend |
| BV421 | CD3ε | UCHT1 | Biolegend |
| Viobright515 | CD56 | REA196 | Miltenyi |
| PE | CD8 | REA734 | Miltenyi |
| PE-Vio770 | CD19 | REA675 | Miltenyi |
| APC | CD4 | OKT4 | Biolegend |
| APC-Vio770 | CD45 | REA747 | Miltenyi |
| PE | CXCR3 | REA232 | Miltenyi |

| | | | |
|---|---|---|---|
| APC | CCR6 | REA190 | Miltenyi |
| BV421 | CD16 | 3G8 | Biolegend |
| FITC | HLA-DR | REA805 | Miltenyi |
| PercP-Vio700 | CD14 | REA599 | Miltenyi |
| PE | CD163 | GHI/61 | Biolegend |
| PE-Cy7 | CCR2 | K036C2 | Biolegend |
| APC | CD86 | IT2.2 | Biolegend |
| AF647 | IL-32 | 373821 | R&D systems |
| BV605 | CD56 | HCD56 | Biolegend |
| BV605 | CD14 | M5E2 | Biolegend |
| BV605 | CD19 | HIB19 | Biolegend |

**Table S4: List of oligonucleotides used for qPCR**

F: forward; R: reverse

| **Primer name** | **Primer sequence** |
|---|---|
| *GAPDH* F | AAGGTCGGAGTCAACGGATTT |
| *GAPDH* R | ATGAAGGGGTCATTGATGGCA |
| *GATA3* F | GCTTCACAATATTAACAGACCCCT |
| *GATA3* R | AAGTCCTCCAGTGAGTCATGC |
| *IL-32* tot F | GATGGATTACGGTGCCGAG |
| *IL-32* tot R | CACAAAAGCTCTCCCCAGG |
| *IL-32G* F | AGGCCCGAATGGTAATGCT |
| *IL-32G* R | CCACAGTGTCCTCAGTGTCACA |
| *TBX21* F | CGCCAGGAAGTTTCATTTGGG |
| *TBX21* R | GAGGGACTGGAGCACAATCAT |

**Table S5: List of antibodies used for immunofluorescence**

| Protein | AB | Host | Company | Application/Dilution |
|---|---|---|---|---|
| **COL1A1** | Monoclonal, #84336 | Rabbit | Cell Signaling | IF; 1:200 |
| **IL-32** | Monoclonal, #MAB4690 | Rabbit | R&D Systems | IF; 1:50 |
| **CD45** | Polyclonal, PA596061 | Mouse | Invitrogen | IF; 1:50 |

## Supplementary methods

### Recording of Cardiomyocytes contractility

To perform the contraction analysis, iPSC-CM were dissociated and replated on 35 mm glass bottom-Geltrex coated dishes (MatTek) suitable for the Contractility acquisition System (IonOptix LLC). iPSC-CM were perfused (at 37 ± 1°C) with an extracellular solution containing: 154 mM NaCl, 4 KCl, 5 mM HEPES NaOH, 2 mM CaCl2, 1 mM MgCl2, 5.5 mM glucose; pH=7.35 in presence or absence of 100ng/mL of IL-32. For the recordings, a region of interest was defined in each selected microscope view field comprising a small number of iPSC-CM. Cells were stimulated at a 0.5 Hz frequency and a video-based contractility acquisition system (CytoMotion) was used to capture contractility via real-time contrast analysis of digitized image data in the selected microscope view field. The IonWizard software was used to collect pixel correlation changes and data were analysed with the CytoSolver software.

## References


1. Luo Y, Xu C, Wang B, Niu Q, Su X, Bai Y, et al. Single-cell transcriptomic analysis reveals disparate effector differentiation pathways in human Treg compartment. Nat Commun. 2021 Jun 23;12(1):3913.
2. Zemmour D, Zilionis R, Kiner E, Klein AM, Mathis D, Benoist C. Single-cell gene expression reveals a landscape of regulatory T cell phenotypes shaped by the TCR. Nat Immunol. 2018 Mar;19(3):291–301.
3. Pesenacker AM, Wang AY, Singh A, Gillies J, Kim Y, Piccirillo CA, et al. A Regulatory T-Cell Gene Signature Is a Specific and Sensitive Biomarker to Identify Children With New-Onset Type 1 Diabetes. Diabetes. 2016 Apr;65(4):1031–9.
4. Galván-Peña S, Leon J, Chowdhary K, Michelson DA, Vijaykumar B, Yang L, et al. Profound Treg perturbations correlate with COVID-19 severity. Proc Natl Acad Sci U S A. 2021 Sep 14;118(37):e2111315118.
5. Luoma AM, Suo S, Wang Y, Gunasti L, Porter CBM, Nabilsi N, et al. Tissue-resident memory and circulating T cells are early responders to pre-surgical cancer immunotherapy. Cell. 2022 Aug 4;185(16):2918-2935.e29.
6. Alshoubaki YK, Nayer B, Lu YZ, Salimova E, Lau SN, Tan JL, et al. Tregs delivered post-myocardial infarction adopt an injury-specific phenotype promoting cardiac repair via macrophages in mice. Nat Commun. 2024 Aug 1;15(1):6480.
7. Schmiedel BJ, Singh D, Madrigal A, Valdovino-Gonzalez AG, White BM, Zapardiel-Gonzalo J, et al. Impact of Genetic Polymorphisms on Human Immune Cell Gene Expression. Cell. 2018 Nov 29;175(6):1701-1715.e16.
8. Reichart D, Lindberg EL, Maatz H, Miranda AMA, Viveiros A, Shvetsov N, et al. Pathogenic variants damage cell composition and single cell transcription in cardiomyopathies. Science. 2022 Aug 5;377(6606):eabo1984.
9. Program CSCB, Abdulla S, Aevermann B, Assis P, Badajoz S, Bell SM, et al. CZ CELL×GENE Discover: A single-cell data platform for scalable exploration, analysis and modeling of aggregated data [Internet]. bioRxiv; 2023 [cited 2024 Oct 17]. p. 2023.10.30.563174. Available from: https://www.biorxiv.org/content/10.1101/2023.10.30.563174v1
